# Genetics, Receptor Binding Property, and Transmissibility in Mammals of Naturally Isolated H9N2 Avian Influenza Viruses

**DOI:** 10.1371/journal.ppat.1004508

**Published:** 2014-11-20

**Authors:** Xuyong Li, Jianzhong Shi, Jing Guo, Guohua Deng, Qianyi Zhang, Jinliang Wang, Xijun He, Kaicheng Wang, Jiming Chen, Yuanyuan Li, Jun Fan, Huiui Kong, Chunyang Gu, Yuantao Guan, Yasuo Suzuki, Yoshihiro Kawaoka, Liling Liu, Yongping Jiang, Guobin Tian, Yanbing Li, Zhigao Bu, Hualan Chen

**Affiliations:** 1 State Key Laboratory of Veterinary Biotechnology, Harbin Veterinary Research Institute, Chinese Academy of Agricultural Sciences, Harbin, People's Republic of China; 2 State Key Laboratory of Veterinary Etiological Biology, Lanzhou Veterinary Research Institute, Chinese Academy of Agricultural Sciences, Lanzhou, People's Republic of China; 3 Laboratory of Avian Disease Surveillance, China Animal Health and Epidemiology Center, Qingdao, People's Republic of China; 4 College of Life and Health Sciences, Chubu University, Aichi, Japan; 5 International Research Center for Infectious Diseases, Institute of Medical Science, University of Tokyo, Tokyo, Japan; National Institutes of Health, United States of America

## Abstract

H9N2 subtype influenza viruses have been detected in different species of wild birds and domestic poultry in many countries for several decades. Because these viruses are of low pathogenicity in poultry, their eradication is not a priority for animal disease control in many countries, which has allowed them to continue to evolve and spread. Here, we characterized the genetic variation, receptor-binding specificity, replication capability, and transmission in mammals of a series of H9N2 influenza viruses that were detected in live poultry markets in southern China between 2009 and 2013. Thirty-five viruses represented 17 genotypes on the basis of genomic diversity, and one specific “internal-gene-combination” predominated among the H9N2 viruses. This gene combination was also present in the H7N9 and H10N8 viruses that have infected humans in China. All of the 35 viruses preferentially bound to the human-like receptor, although two also retained the ability to bind to the avian-like receptor. Six of nine viruses tested were transmissible in ferrets by respiratory droplet; two were highly transmissible. Some H9N2 viruses readily acquired the 627K or 701N mutation in their PB2 gene upon infection of ferrets, further enhancing their virulence and transmission in mammals. Our study indicates that the widespread dissemination of H9N2 viruses poses a threat to human health not only because of the potential of these viruses to cause an influenza pandemic, but also because they can function as “vehicles” to deliver different subtypes of influenza viruses from avian species to humans.

## Author Summary

Avian influenza viruses continue to present challenges to human health. Recently the H7N9 and H10N8 viruses that are of low pathogenicity for poultry have caused human infections and deaths in China. H9N2 influenza virus have been isolated worldwide from wild and domestic avian species for several decades, and their low pathogenic nature to poultry made them a low priority for animal disease control, which has allowed them to continue to evolve and spread. Here, we investigated a series of H9N2 influenza viruses that were detected in live poultry markets in southern China. We found that these viruses are able to preferentially bind to the human-type receptor, and some of them can cause disease and transmit between ferrets by respiratory droplet. All the transmissible H9N2 viruses have a similar internal gene constellation, which was also present in the H7N9 and H10N8 viruses. Our study indicates that the widespread dissemination of H9N2 viruses poses a threat to human health not only because of the potential of these viruses to cause an influenza pandemic, but also because they can function as “vehicles” to deliver different subtypes of influenza viruses from avian species to humans.

## Introduction

Avian influenza viruses of several subtypes continue to present challenges to human health. The H5N1 influenza viruses have caused 380 fatal cases among the 641 documented human infections in 16 countries [1], and several studies have documented the transmission potential of H5N1 mutants or reassortants [2]–[4]. Eighty-seven human cases of H7N7 influenza virus infection were confirmed in the Netherlands in 2003, one of which was fatal [5], [6]. H6 viruses can infect and cause illness in mice and ferrets, and are transmissible in guinea pigs [7]–[9]; an H6N1 virus was isolated from a human with influenza-like symptoms in Taiwan in 2013 [10]. An approximately 30% mortality rate is associated with the 400 human infections with the newly emerged H7N9 viruses in China reported by the end of March, 2014 [11]. H10N8 virus caused three human infections in China in 2013, two of which were fatal [12]. These facts emphasize that it is not only the highly pathogenic H5N1 and H7N7 influenza viruses that pose a severe threat to human health, but also that the nonlethal influenza viruses circulating in avian species can cause disease and even death in humans.

During the last several decades, H9N2 influenza viruses have been isolated worldwide from wild and domestic avian species [13], [14]. These viruses have also been detected in pigs [15]–[17]. Many studies have been performed to evaluate the pandemic potential of the H9N2 influenza viruses. The viruses have been shown to replicate in mice without pre-adaptation [18]–[21], and some strains from poultry in Asia have human virus-like receptor specificity [22]. Sorrell et al. reported that following adaptation in the ferret, a reassortant carrying the surface proteins of an avian H9N2 in a human H3N2 backbone could transmit efficiently via respiratory droplet [23]. Other studies have reported that H9N2 reassortants bearing genes from the 2009 H1N1 pandemic virus exhibited increased virulence in mice [24] or transmissibility in ferrets [25]. Wan et al. found that two of five wild-type H9N2 viruses isolated from different avian species between 1988 and 2003 transmitted to direct contact ferrets [26]. However, none of the naturally isolated H9N2 viruses has been reported to transmit to ferrets via respiratory droplet.

Although the H9N2 viruses have been detected in chickens and ducks in many provinces in China since 1993 [18], [27], their low pathogenic nature to poultry has made them a low priority for animal disease control. However, the H9N2 viruses caused human infections in China in 1999, 2003, and 2013 [28]–[30], and some poultry workers in China, India, Cambodia, Romania, America, Nigeria, and Vietnam were reportedly serologically positive for H9N2 viruses [31]–[38], implying a substantial threat to public health. Recent studies indicated that the H9N2 viruses contributed the six internal genes to the newly emerged H7N9 virus in southern China and to the H10N8 virus that caused three human infections in Jiangxi province, China [12], [39], [40]. These facts prompted us to assess the biologic properties and pandemic potential of H9N2 influenza viruses circulating in poultry.

## Results

### Genetic characterization of H9N2 influenza viruses isolated in poultry between 2009 and 2013

**Figure 1:**
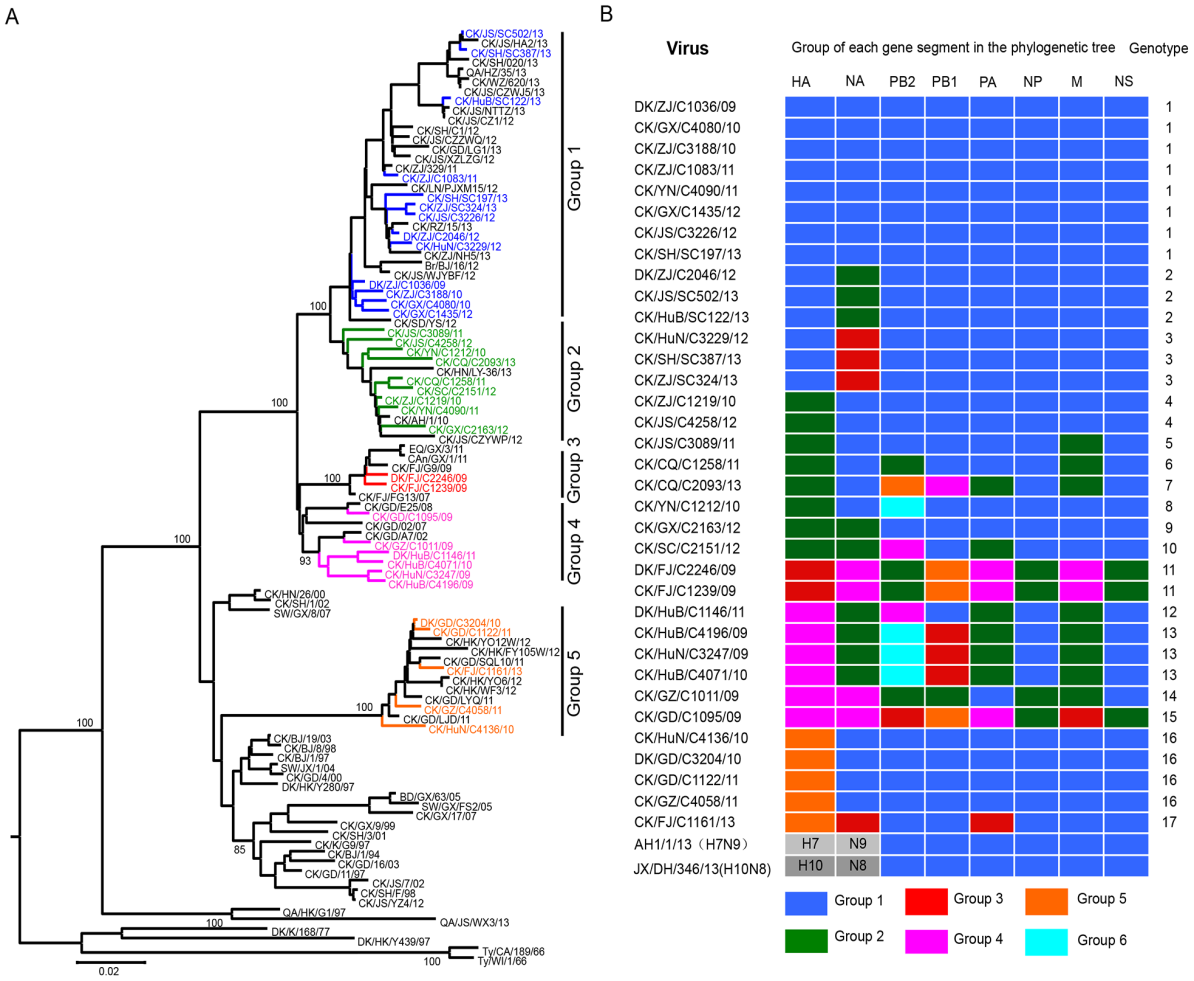
Genetic relationships among the HA genes and genotype evolution of H9N2 viruses. (**A**) Phylogenetic tree of HA. The tree was based on nucleotides (nt) 128 to 1540 and rooted to A/Duck/Alberta/60/76(H12N5). Sequences of viruses with names in black were downloaded from available databases; viruses with names in other colors were sequenced in this study. Abbreviations are as follows: Br, Brambling; CAn, Canine; CK, Chicken; DK, Duck; Env, Environment; GS, Goose; Pg, Pigeon; QA, Quail; SW, Swine; Ty, Turkey; AH, Anhui; BJ, Beijing; CA, California; CQ, Chongqing; DH, Donghu; FJ, Fujian; GD, Guangdong; GX, Guangxi; GZ, Guangzhou; HZ, Hangzhou; HN, Henan; HK, Hong Kong; HuB, Hubei; HuN, Hunan; JS, Jiangsu; SD, Shandong; SH, Shanghai; TZ, Taizhou; WI, Wisconsin; XZ, Xuzhou; YZ, Yangzhou; YN, Yunnan; ZJ, Zhejiang. (**B**) Genotypes of the H9N2 viruses. The eight gene segments are indicated at the top of each bar.

To investigate the genetic relationship of the viruses from different times and places, we sequenced the genomes of 35 viruses that were collected from 2009 to 2013 from 12 provinces in southern China (Figure S1). The amino acid motif at the cleavage site of the hemagglutinin (HA) of these isolates is –RSSR-, which is a characteristic of viruses of low pathogenicity in chickens. The HA gene of the 35 viruses shared 87.8%–99.7% identity at the nucleotide level, and they formed five phylogenetic groups (Figure 1A). The neuraminidase (NA) genes of these viruses shared 85.4%–99.6% identity at the nucleotide level and formed four phylogenetic groups (Figure S2A). The 31 viruses in groups 1, 2 and 3 have a 3-amino acid deletion in the NA stalk (residues 62–64), whereas the four isolates in group 4 have no such deletion.

Several amino acid changes related to the increased replication or virulence of avian influenza viruses in mammals [41] were detected in these H9N2 viruses (Table S1). The amino acid changes R207K, H436Y, and M677T in basic polymerase 1 (PB1) [42], [43], A515T in acidic polymerase (PA) [42], N30D and T215A in matrix protein (M1) [44], and P42S in nonstructural protein 1 (NS1) were conserved in all strains [45], whereas the virulence-related mutation I368V in PB1[3], T139A in M1[46], and the amantadine and rimantadine resistance-conferring mutation S31N/G in M2 were detected in some of the strains (Table S1) [47]. Two amino acid changes in basic polymerase 2 (PB2), glutamic acid to lysine at position 627(E627K) and aspartic acid to asparagine at position 701 (D701N), are important for the virulence and transmission of H5N1 viruses in mammals [48]–[50], and are also frequently presented in the H7N9 viruses isolated from humans [40], [51]. A detailed comparison of the amino acid differences among the H9N2 viruses showed that all 35 of the H9N2 viruses have the amino acid combination of 627E/701D in their PB2.

The six internal genes of the H9N2 viruses showed distinct diversity, with PB2, PB1, PA, nucleoprotein (NP), M, NS genes of the 35 viruses sharing 84.8%–99.4%, 86.4%–99.6%, 87.9%–99.5%, 92.5%–99.6%, 94.1%–99.9%, and 91.3%–99.6% identity, respectively, at the nucleotide level. The PB2 genes formed six groups in their polygenic trees (Figure S2B), and the PB1 genes formed five groups in their polygenic trees (Figure S2C). The PA and M genes each formed four groups in their polygenic trees (Figure S2D and Figure S2F), whereas the NP and NS genes each formed two groups in their polygenic trees (Figure S2E and Figure S2G).

On the basis of this genomic diversity, the viruses examined in this study were divided into 17 genotypes (Figure 1B). Of note, 19 viruses in genotypes 1, 2, 3, 4, and 16 have a similar combination of their six internal genes (the DK/ZJ/C1036/09-like combination).

### Receptor-binding preference of the H9N2 influenza viruses

**Figure 2:**
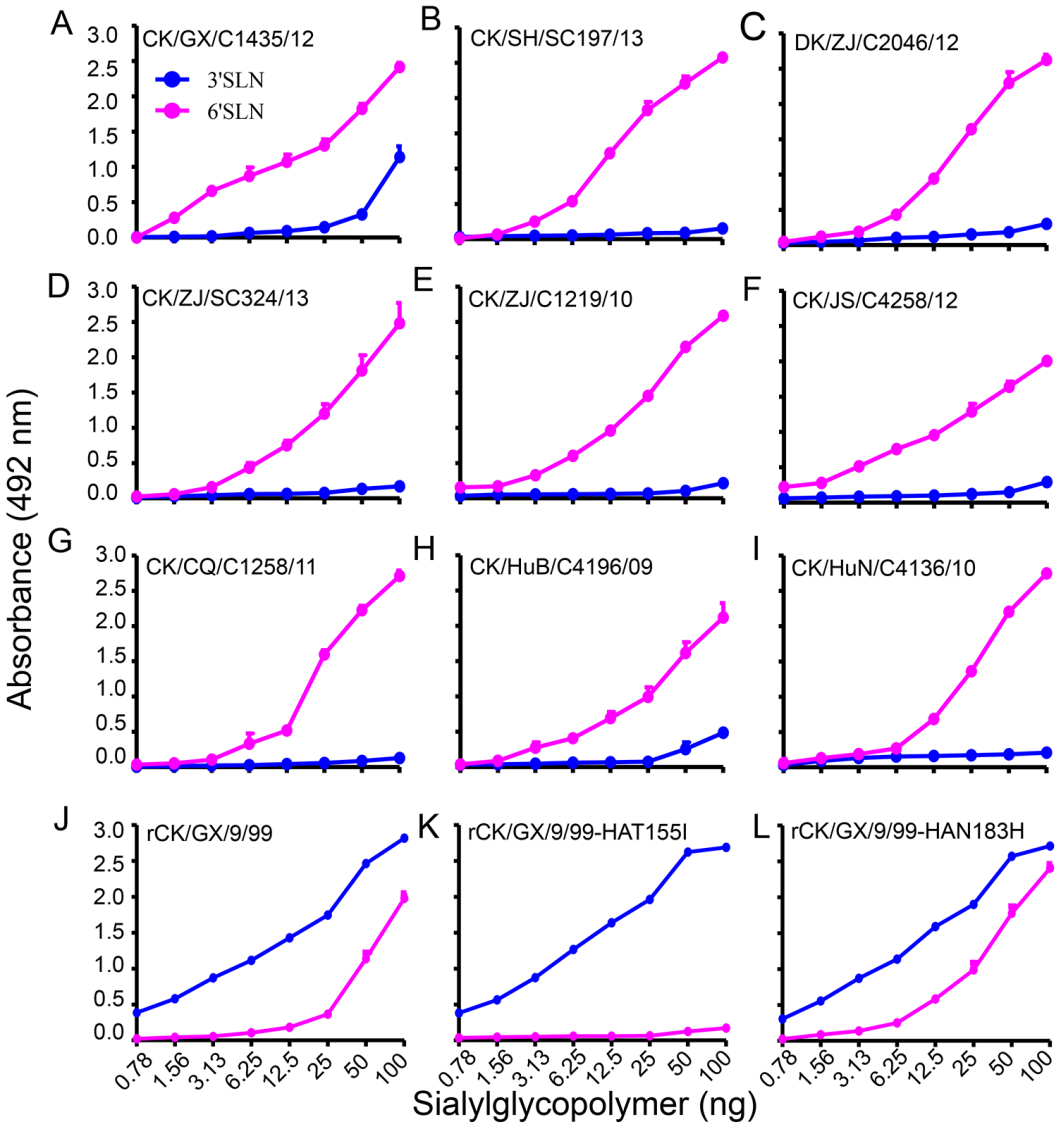
Characterization of the receptor-binding properties of H9N2 viruses. The binding of the viruses to two different biotinylated glycans (α-2, 3 glycan, blue; α-2, 6 glycan, pink) was tested. The data shown are the means of three repeats; the error bars indicate the standard deviations. (**A**) CK/GX/C1435/12. (**B**) CK/SH/SC197/13. (**C**) DK/ZJ/C2046/12. (**D**) CK/ZJ/SC324/13. (**E**) CK/ZJ/C1219/10. (**F**) CK/JS/C4258/12. (**G**) CK/CQ/C1258/11. (**H**) CK/HuB/C4196/09. (**I**) CK/HuN/C4136/10. (**J**) rCK/GX/9/99. (**K**) rCK/GX/9/99-HAT155I. (**L**) rCK/GX/9/99-HAN183H.

Receptor-binding preference has important implications for influenza virus replication and transmission [3], [4]. The change of receptor-binding preference from α-2, 3-linked sialic acids (Sias) (avian-type receptors) to α-2, 6-linked Sias (human-type receptors) is thought to be a prerequisite for an avian influenza virus to transmit from human to human. By using a solid-phase binding assay as described previously [4], [7], we tested the receptor-binding specificity of 41 H9N2 viruses, the 35 viruses described above and six "early" viruses that were isolated from poultry in China between 1996 and 2001 [18] (Figure 2, Figure S3), to two different glycopolymers: the α-2, 3-siaylglycopolymer [Neu5Acα2-3Galβ1-4GlcNAcβ1-pAP (para-aminophenyl)-alpha-polyglutamic acid (α-PGA)] and the α-2, 6-sialylglycopolymer [Neu5Acα2-6Galβ1-4GlcNAcβ1-pAP (para-aminophenyl)-alpha-polyglutamic acid (α-PGA)]. All 41 viruses were able to bind to the α-2, 6-siaylglycopolymer, although eight, including the six "early" viruses, also bound to the α-2, 3-siaylglycopolymer with moderate to high affinity (Figure 2, Figure S3, and Table S2). These results indicate that H9N2 viruses isolated naturally from poultry have acquired the ability to preferentially bind to the human-type receptor, similar to the widely circulating human influenza viruses.

### The amino acid mutation I155T (H3 numbering used throughout) in HA favors the binding of H9N2 virus to the α-2, 6-siaylglycopolymer

The amino acid change Q226L is reported to contribute to the human-type receptor binding of H9N2 virus [26], but this mutation was not detected in the six "early" isolates in our study (Table S1), which were able to bind to the α-2, 6-siaylglycopolymer (Figure S3), suggesting that some other amino acid(s) may contribute to this phenotype. As shown in Table S1, three more amino acid changes (I155T, H183N, and A190V) that have been reported to affect the receptor binding preference of other subtypes of influenza viruses were also detected in these H9N2 viruses. Since the amino acid at position 190 of HA was not conserved, and this amino acid has not been linked to the receptor-binding phenotype observed, we investigated the contributions of only I155T and H183N to the human-type receptor binding of H9N2 virus.

By using plasmid-based reverse genetics, we constructed a reassortant virus containing the HA and NA genes of the "early" H9N2 isolate A/chicken/Guangxi/9/99 (CK/GX/9/99) and the six internal genes of the A/Puerto Rico/8/1934 (H1N1) (PR8) virus and designated it as rCK/GX/9/99. Then, we introduced the avian influenza virus-like amino acids I and H into the HA gene at positions 155 and 183, respectively, to create the mutants we designated as rCK/GX/9/99-HAT155I and rCK/GX/9/99-HAN183H, respectively. Receptor binding analysis indicated that, similar to the wild-type CK/GX/9/99 virus (Figure S3), the rCK/GX/9/99 and rCK/GX/9/99-HAN183H viruses bound to both the α-2, 3-siaylglycopolymer and α-2, 6-siaylglycopolymer (Figure 2J and L); however, the rCK/GX/9/99-HAT155I variant only maintained the ability to bind to the α-2, 3-siaylglycopolymer and lost its ability to bind to the α-2, 6-siaylglycopolymer (Figure 2K). These results indicate that, in addition to the Q226L mutation, the I155T mutation in HA also plays an important role in the binding of H9N2 virus to the human-type receptor.

### Replication and virulence of the H9N2 viruses in mice

**Figure 3:**
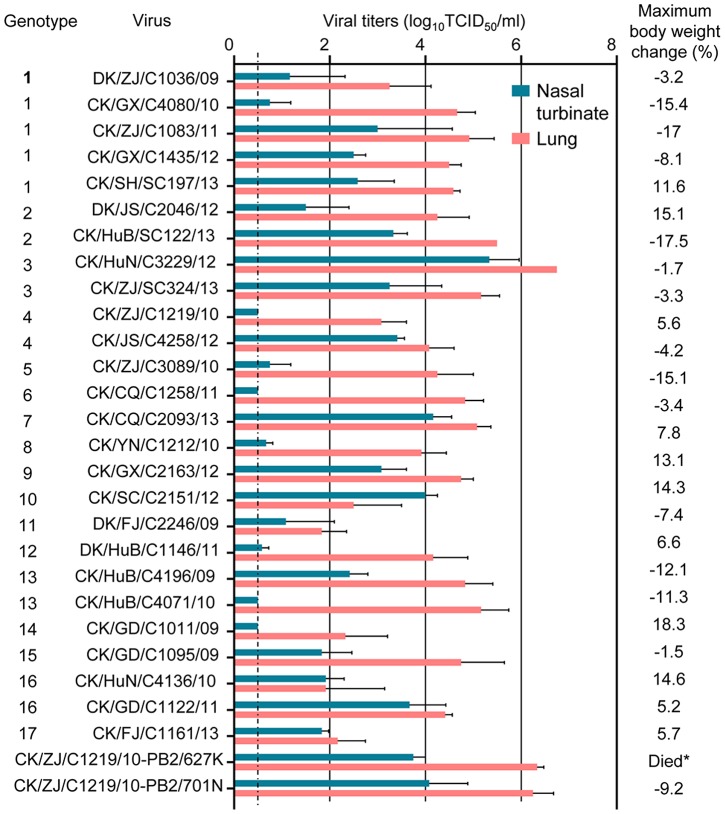
Replication and virulence of H9N2 viruses in mice. Virus titers in organs of mice on day 3 p.i. with 10^6^ EID_50_ of test virus. Data shown are the mean titers from three mice; the error bars indicate the standard deviations. *, mice inoculated with CK/ZJ/C1219/10-PB2/627K virus died before day 8 p.i. The dashed line indicates the lower limit of detection.

We selected 26 H9N2 influenza viruses, to include one virus from each genotype from each year, and evaluated their replication and virulence in BALB/c mice. All 26 viruses replicated in lungs of mice, with titers ranging from 1.8 to 6.8log_10_TCID_50_, 22 viruses were also detected in the nasal turbinates of mice, with titers ranging from 0.7 to 5.3log_10_TCID_50_ (Figure 3). Virus was not detected in the spleen, kidneys, or brain of any mice. Mice infected with these viruses showed diverse body weight changes during the observation period: fourteen viruses caused 1.5% to 17.5% body weight loss in mice, whereas mice gained body weight despite inoculation with the other twelve viruses (Figure 3). All mice survived during the observation period. These results indicate that, unlike the H7N9 viruses isolated from poultry, which did not cause any disease in mice [40], the H9N2 viruses isolated from poultry show a range of virulence in mice.

### Replication of H9N2 viruses in ferrets

As shown in Figure 1B and Table S3, the viruses of genotypes 1–4, 13, and 16 were detected from multiple provinces and in different years, and we therefore selected one or two viruses from these genotypes and tested their replication and transmission in ferrets. However, the viruses of genotypes 5–12, 14, 15, and 17 were only isolated from individual provinces; therefore, we speculated that those strains were unlikely to be widespread and, accordingly, only one strain from genotype 6 was selected and tested in ferrets (Table S3).

**Figure 4:**
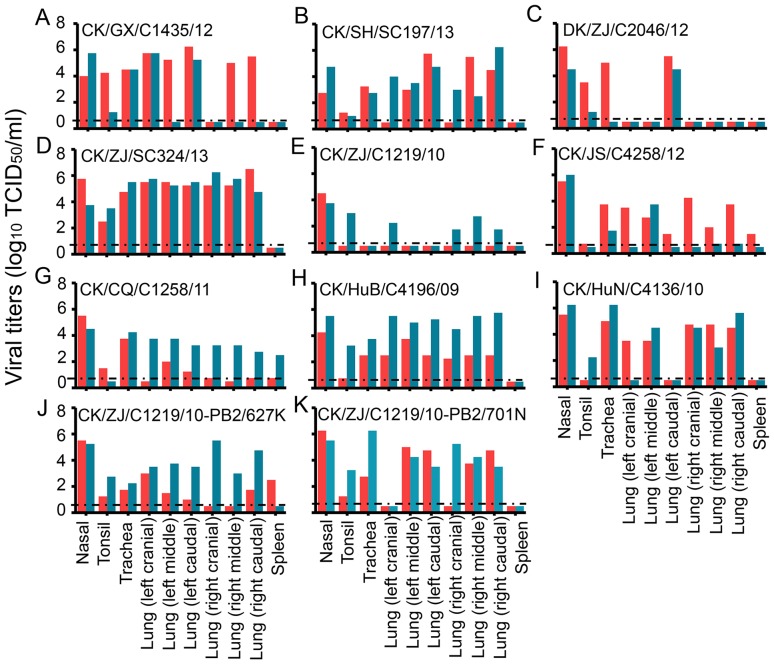
Replication of H9N2 viruses in ferrets. (**A**) CK/GX/C1435/12. (**B**) CK/SH/SC197/13. (**C**) DK/ZJ/C2046/12. (**D**) CK/ZJ/SC324/13. (**E**) CK/ZJ/C1219/10. (**F**) CK/JS/C4258/12. (**G**) CK/CQ/C1258/11. (**H**) CK/HuB/C4196/09. (**I**) CK/HuN/C4136/10. (**J**) CK/ZJ/C1219/10-PB2/627K. (**K**) CK/ZJ/C1219/10-PB2/701N. Each color bar represents the virus titer from an individual animal. The dashed black lines indicate the lower limit of detection.

Two ferrets were inoculated i.n. with 10^6^ EID_50_ of each virus, and the nasal turbinates, tonsils, trachea, various lung lobes, brain, spleen, kidneys, and liver from each ferret were collected on day 4 p.i. for virus titration in MDCK cells. All of the nine viruses replicated in the nasal turbinates of ferrets, with titers ranging from 2.8–7.3log_10_TCID_50_ (Figure 4). Virus replication in trachea was detected in ferrets inoculated by eight viruses, but not in the CK/ZJ/C1219/10 virus-infected ferrets (Figure 4E). Virus was detected in all lobes of lungs of ferrets inoculated with CK/ZJ/SC324/13 and CK/HuB/C4196/09 (Figure 4D and H), but was not detectable in some of lobes of the lungs of ferrets inoculated with the other seven viruses (Figure 4). Virus was detected in the spleen of one ferret inoculated with CK/JS/C4258/12 and two ferrets inoculated with CK/CQ/C1258/11(Figure 4F and G), but was not detected in the brain, kidney, or liver of any ferret.

**Figure 5:**
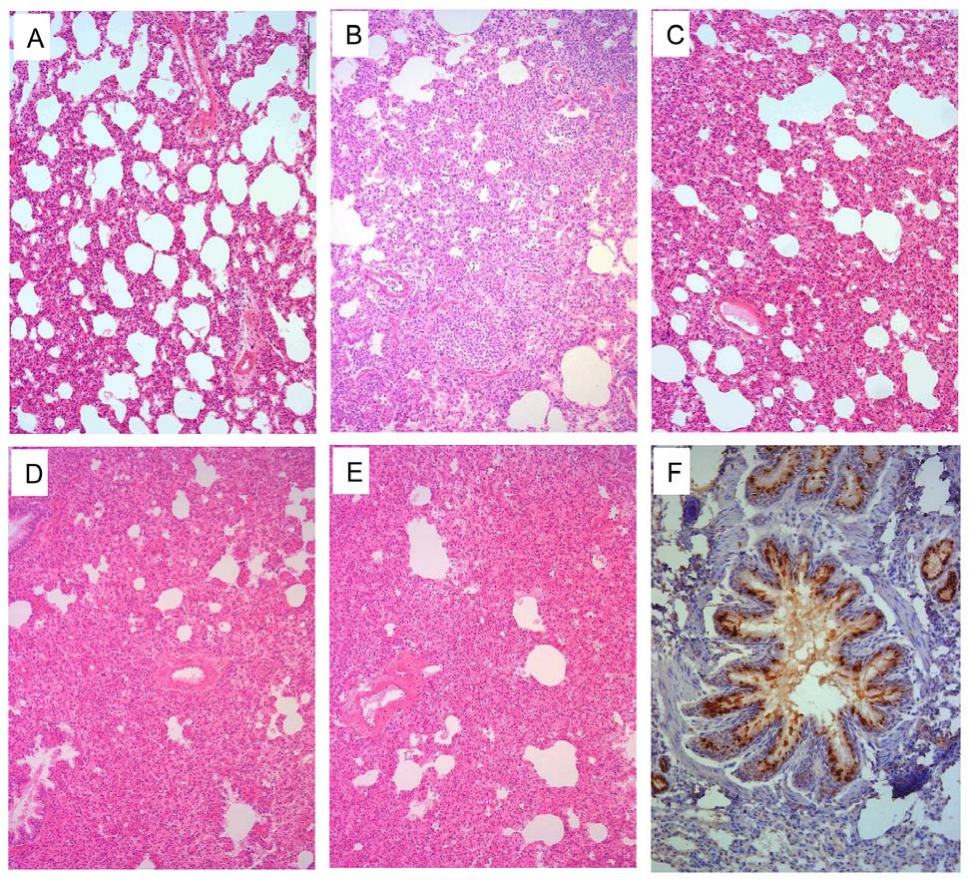
Histological lesions caused by H9N2 viruses in the lungs of ferrets. Ferrets were euthanized on day 4 p.i. with 10^6^EID_50_ of test virus, and the lungs were collected for pathological study. The lungs of CK/ZJ/C1219/10 virus-inoculated animal showed only mild histopathological changes (H&E staining,) (**A**), whereas the lungs of CK/GX/C1435/12 (**B**), CK/JS/C4258/12 (**C**), CK/ZJ/C1219/10-PB2/627K (**D**), and CK/ZJ/C1219/10-PB2/701N (**E**) virus-inoculated ferrets showed severe pathological lesions (H&E staining) Viral antigen was detected in the epithelial cells of bronchus and alveoli by means of immunohistochemical (IHC) staining (**F**, from the lung samples of a ferret inoculated with CK/JS/C4258/12 virus). Images **A–E** were taken at ×100 magnification; Image **F** was taken at ×200 magnification.

Pathological studies were performed on lung samples from the virus-infected ferrets. Most of the lungs showed mild damage after infection with CK/ZJ/C1219/10 or DK/ZJ/C2046/12 (Figure 5A, Figure S4A). By contrast, the lungs of the other seven virus-infected ferrets showed severe bronchopneumonia (Figure 5 B and C, and Figure S4B–F).

### Transmission of H9N2 influenza viruses between ferrets

To investigate respiratory droplet transmission, we inoculated three ferrets i.n. with 10^6.0^ EID_50_ of test virus and then housed them separately in solid stainless-steel cages within an isolator. Twenty-four hours later, three naïve ferrets were placed in adjacent cages. Each pair of animals was separated by a double-layered net divider as described previously [2], [40]. Nasal washes were collected every 2 days from all of the animals beginning 2 days p.i. [1 day post-exposure (p.e.)] for the detection of virus shedding. Sera were collected from all animals on day 21 p.i. for hemagglutinin inhibition (HI) antibody detection. Respiratory droplet transmission was confirmed when virus was detected in the nasal washes or by seroconversion of the naïve exposed animals at the end of the 3-week observation period.

**Figure 6:**
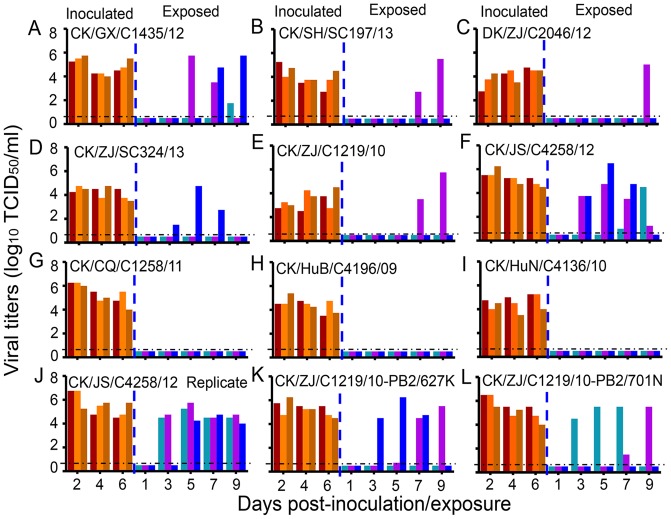
Respiratory droplet transmission of H9N2 viruses in ferrets. (**A**) CK/GX/C1435/12. (**B**) CK/SH/SC197/13. (**C**) DK/ZJ/C2046/12. (**D**) CK/ZJ/SC324/13. (**E**) CK/ZJ/C1219/10. (**F**) CK/JS/C4258/12. (**G**) CK/CQ/C1258/11. (**H**) CK/HuB/C4196/09. (**I**) CK/HuN/C4136/10. (**J**) CK/JS/C4258/12 (replicate). (**K**) CK/ZJ/C1219/10-PB2/627K. (**L**) CK/ZJ/C1219/10-PB2/701N. Each color bar represents the virus titer from an individual animal. The dashed black lines indicate the lower limit of detection.

Virus was detected in all of the directly infected animals (Figure 6A–L). However, virus was not detected in any of the animals exposed to the CK/CQ/C1258/11-, CK/HuB/C4196/09-, and CK/HuN/C4136/10-inoculated ferrets (Figure 6G, H, and I). Virus was detected in one ferret exposed to the ferrets that had been inoculated with the CK/SH/SC197/13, DK/JS/C2046/12, CK/ZJ/SC324/13, and CK/ZJ/C1219/10 viruses (Figure 6B, C, D, and E). Virus was detected in all three ferrets exposed to the ferrets that had been inoculated with CK/GX/C1435/12 and CK/JS/C4258/12 (Figure 6A and F). Because the efficient transmission of naturally isolated H9N2 influenza viruses has never been reported before, we repeated this respiratory droplet transmission study with the CK/JS/C4258/12 virus in ferrets and found the results to be reproducible (Figure 6J). The ferrets that were inoculated with these nine viruses experienced a 1.4% to 7.9% body weight loss, and the body weight loss of the exposed ferrets was up to 8.8% (Table 1 and Table S4). Seroconversion occurred in all of the virus-inoculated animals and in all exposed animals that were virus-positive (Table 1). These results indicate that six of the nine H9N2 viruses tested can transmit between ferrets, and two of them transmit highly efficiently via respiratory droplet.

**Table 1:** Virus replication in and seroconversion of ferrets inoculated with or exposed to different H9N2 influenza viruses.

Virus (Genotype)	Maximum body temperature increase (°C)^a^		Maximum body weight loss (%)^a^		Seroconversion:positive/total (HI antibody titer range)^b^	
	Inoculated	Exposed	Inoculated	Exposed	Inoculated	Exposed
CK/GX/C1435/12 (1)	2	3.4	7.9	5.2	3/3 (320–640)	3/3 (640)
CK/SH/SC197/13 (1)	1.5	1.3	3.1	3.3	3/3 (640–2560)	1/3(1280)
DK/ZJ/C2046/12 (2)	2.4	0	5.2	2.9	3/3 (2560–5120)	1/3 (2560)
CK/ZJ/SC324/13 (3)	1.7	1.3	2.6	0.9	3/3 (640–1280)	1/3(640)
CK/ZJ/C1219/10 (4)	0.9	1.5	1.4	1.8	3/3 (1280–2560)	1/3 (640)
CK/JS/C4258/12 (4) c	1.9	2.4	3.3	8.8	6/6 (640–1280)	6/6 (320–1280)
CK/CQ/C1258/11 (6)	2.5	0.5	2.7	0.3	3/3 (40–320)	0/3
CK/HuB/C4196/09 (13)	1.2	0	3.9	0	3/3 (320–1280)	0/3
CK/HuN/C4136/10 (16)	1.1	0	4.2	0.5	3/3 (1280–2560)	0/3
CK/ZJ/C1219/10-PB2/627K (4)	1.4	1.3	6.4	4.5	3/3 (1280–2560)	2/3 (2560)
CK/ZJ/C1219/10-PB2/701N (4)	1.2	1.3	7.2	4.4	3/3 (1280–2560)	2/3 (640–1280)

a Data shown are from the animal in that group with the maximum body temperature increase or maximum body weight loss.

b Seroconversion was confirmed from ferret serum that was collected on day 21 post-inoculation.

c The CK/JS/C4258/10 experiment was conducted twice; the combined data are presented.

### The 627K and 701N mutations in PB2 increase the virulence and transmission of H9N2 viruses in mammals

Previous studies showed that the H7N9 viruses easily acquire the 627K or 701N mutations in PB2 during their replication in humans [40], [52], [53]. To investigate whether the H9N2 viruses similarly acquire such mutations during replication in ferrets, we sequenced the PB2 gene of ten randomly selected clones of each sample recovered at different time points from each ferret. The 627K or 701N mutations in PB2 were detected from the samples recovered from the ferrets that were inoculated or exposed to CK/GX/C1435/12, CK/ZJ/C1219/10, CK/CQ/C1258/11, CK/JS/C4258/12, CK/HuB/C4196/09, and CK/HuN/C4136/10, but were not detected in the samples recovered from the ferrets that were inoculated with or exposed to CK/SH/SC197/13, DK/ZJ/C2046/12, and CK/ZJ/SC324/13 viruses (Table 2). To investigate whether these mutations existed in the inoculums, the PB2 gene of all nine viruses tested in ferrets was checked by using a deep sequencing approach; the 627K and 701N mutations in PB2 were not detected in any of the viral stocks (Table 2, Table S5). We also deep sequenced the PB2 of nine selected samples that were recovered from the virus-inoculated ferrets, and found that the ratios of the PB2 627K and 701N mutations were comparable to our previous sequencing results presented in Table 2 (Table S5).

**Table 2:** Assessment of the presence or absence of the 627K and 701N mutations in the PB2 segment of viruses recovered from the nasal washes of H9N2 influenza virus infected or exposed ferrets.

Virus	Stock virus a		Animal pair	Virus recovered from animal on different days post-inoculation (p.i.) or post-exposure (p.e.) b													
				Day 2 p.i.		Day 4 p.i.		Day 6 p.i.		Day 3 p.e.		Day 5 p.e.		Day 7 p.e.		Day 9 p.e.	
	627K	701N		627K	701N	627K	701N	627K	701N	627K	701N	627K	701N	627K	701N	627K	701N
CK/GX/C1435/12	0%	0%	1	0/10	7/10	ND	ND	0/10	8/10	/	/	/	/	/	/	0/10	10/10
			2	2/10	3/10	ND	ND	2/10	7/10	/	/	1/10	0/10	6/10	1/10	/	/
			3	2/10	6/10	ND	ND	0/10	10/10	/	/	/	/	9/10	1/10	ND	ND
CK/SH/SC197/13	0%	0%	1	ND	ND	ND	ND	0/10	0/10	/	/	/	/	/	/	/	/
			2	ND	ND	ND	ND	0/10	0/10	/	/	/	/	0/10	0/10	0/10	0/10
			3	ND	ND	ND	ND	0/10	0/10	/	/	/	/	/	/	/	/
DK/ZJ/C2046/12	0%	0%	1	ND	ND	ND	ND	0/10	0/10	/	/	/	/	/	/	/	/
			2	ND	ND	ND	ND	0/10	0/10	/	/	/	/	/	/	0/10	0/10
			3	ND	ND	ND	ND	0/10	0/10	/	/	/	/	/	/	/	/
CK/ZJ/SC324/13	0%	0%	1	ND	ND	ND	ND	0/10	0/10	/	/	/	/	/	/	/	/
			2	ND	ND	ND	ND	0/10	0/10	/	/	/	/	/	/	/	/
			3	ND	ND	ND	ND	0/10	0/10	/	/	0/10	0/10	0/10	0/10	/	/
CK/ZJ/C1219/10	0%	0%	1	ND	ND	0/10	0/10	1/10	5/10	/	/	/	/	/	/	/	/
			2	ND	ND	0/10	4/10	0/10	8/10	/	/	/	/	0/10	9/10	0/10	10/10
			3	ND	ND	0/10	1/10	0/10	6/10	/	/	/	/	/	/	/	/
CK/JS/C4258/12	0%	0%	1	0/10	0/10	ND	ND	0/10	6/10	/	/	/	/	ND	ND	0/10	0/10
			2	0/10	0/10	ND	ND	0/10	6/10	ND	ND	0/10	0/10	ND	ND	0/10	0/10
			3	0/10	1/10	ND	ND	1/10	7/10	0/10	4/10	0/10	1/10	0/10	6/10	/	/
CK/JS/C4258/12 (Replicate)	0%	0%	1	0/10	2/10	ND	ND	0/10	8/10	0/10	10/10	0/10	10/10	ND	ND	0/10	10/10
			2	0/10	0/10	8/10	0/10	10/10	0/10	0/10	8/10	0/10	10/10	ND	ND	0/10	10/10
			3	0/10	0/10	0/10	6/10	0/10	10/10	/	/	0/10	0/10	ND	ND	0/10	0/10
CK/CQ/C1258/11	0%	0%	1	ND	ND	0/10	0/10	0/10	1/10	/	/	/	/	/	/	/	/
			2	ND	ND	0/10	0/10	0/10	0/10	/	/	/	/	/	/	/	/
			3	ND	ND	0/10	0/10	0/10	1/10	/	/	/	/	/	/	/	/
CK/HuB/C4196/09	0%	0%	1	ND	ND	0/10	2/10	0/10	2/10	/	/	/	/	/	/	/	/
			2	ND	ND	0/10	1/10	0/10	1/10	/	/	/	/	/	/	/	/
			3	ND	ND	0/10	3/10	0/10	1/10	/	/	/	/	/	/	/	/
CK/HuN/C4136/10	0%	0%	1	ND	ND	ND	ND	0/10	4/10	/	/	/	/	/	/	/	/
			2	ND	ND	ND	ND	0/10	0/10	/	/	/	/	/	/	/	/
			3	ND	ND	ND	ND	0/10	0/10	/	/	/	/	/	/	/	/

a Results were obtained by using a deep sequencing approach.

b We extracted viral RNA from nasal washes collected from virus-inoculated and virus-exposed ferrets. PB2 and HA were amplified and cloned into T vectors. Ten molecular clones from each sample were randomly selected and sequenced. No changes in HA were detected. For values in parentheses, the number on the left of the slash shows the number of clones bearing the indicated amino acid at position 627 or 701, and the number on the right of the slash shows the total number of clones sequenced. **ND**, not done; **/**, not applicable.

To investigate whether the 627K or 701N mutations in PB2 could increase the virulence and transmissibility of the H9N2 viruses in mammals, we plaque-purified two mutants, CK/ZJ/C1219/10-PB2/627K and CK/ZJ/C1219/10-PB2/701N, and tested them in mice and ferrets. The viral titers in the nasal turbinates and lungs of the mutant-infected mice were significantly higher than that of the CK/ZJ/C1219/10 virus-inoculated mice (Figure 3). The CK/ZJ/C1219/10-PB2/627K virus killed all five mice by day 6 p.i., whereas the mice inoculated with the CK/ZJ/C1219/10-PB2/701N virus experienced a 9.2% body weight loss and survived the infection for the observation period (Figure 3). Viral titers in the nasal turbinates and lungs of the two mutant-inoculated ferrets were notably higher than that of the CK/ZJ/C1219/10-inoculated ferrets, and virus was also detected in the spleen of one ferret inoculated with the CK/ZJ/C1219/10-PB2/627K virus (Figure 4, J and K). The lung damage of the two mutant-inoculated ferrets was much more severe than that of the CK/ZJ/C1219/10-inoculated ferrets (Figure 5, D and E). Both mutants transmitted to two of three ferrets via respiratory droplet (Figure 6, K and L, Table 1). The CK/ZJ/C1219/10-PB2/627K-inoculated and -exposed ferrets experienced 6.4% and 4.5% weight loss, respectively, and the body weight loss of the CK/ZJ/C1219/10-PB2/701N-inoculated and -exposed ferrets was 7.2% and 4.4%, respectively (Table 1, Table S4). These results indicate that the 627K and 701N mutations in PB2 increase the virulence and further promote the transmissibility of H9N2 viruses in mammals.

## Discussion

Receptor-binding preference has important implications for influenza virus replication and transmission [3], [4], [54], [55]. Generally, it is believed that the HA of human infective influenza subtypes preferentially recognizes α-2, 6- linked Sias, whereas the HA of avian influenza subtypes preferentially recognizes α-2, 3-linked Sias [3], [56]. Some naturally isolated avian influenza viruses of the H5 and H6 subtypes have been reported to bind to α-2, 6-linked Sias [2], [7], [50], but their affinity to the α-2, 3-linked Sias was much higher than that to α-2, 6-linked Sias. The newly emerged H7N9 viruses isolated from both avian species and humans bind to α-2, 6-linked Sias with high affinity and to α-2, 3-linked Sias with affinity that varies among strains [40], [57]. However, most of the H9N2 viruses circulating in China bind exclusively to α-2, 6-linked Sias, as is observed with human influenza viruses. Thus influenza viruses can acquire the ability to bind human-type receptors during their circulation in avian species, and mammalian intermediate hosts, for example pigs, are not necessarily needed for this process.

The molecular determinants of the receptor binding preference of influenza viruses are not fully understood. Several amino acid changes in HA, including I155T, H183N, A190V, Q226L, and G228S, have been reported to promote the affinity of avian influenza viruses for human-type receptors [22], [50], [54], [58], [59]. Although 155T and 183N were conserved in all of the H9N2 viruses (Table S1), our mutagenesis study indicated that 155T, but not 183N, is necessary for the H9N2 virus to bind to the human-type receptor. The amino acid at 190 was not conserved in these strains, and the avian influenza virus-like 190A was present in several viruses that exclusively bound to the α-2,6-linked Sias; the amino acid change G228S was not detected in any of our H9N2 viruses. Therefore, the H183N, A190V, and G228S mutations in HA are not necessary for an H9N2 virus to bind to the human-type receptor, whereas the mutations I155T and Q226L play important roles in H9N2 virus binding to the human-type receptor.

Avian influenza viruses can acquire different mutations that confer increased receptor-binding ability, virulence, or transmissibility during their replication in mammalian hosts, and some of these mutations have been detected in the H9N2 viruses (Table S1), although we were not able to find a strong relationship between these changes and the observed virulence or transmissibility in mammals of our H9N2 viruses. The 627K and 701N mutations in PB2 were detected in some viruses recovered from both inoculated and exposed ferrets, indicating that certain H9N2 viruses are predisposed to acquiring the 627K or 701N mutation in their PB2 gene when they replicate in mammals. However, the absence of the 627K and 701N mutations in the PB2 of some of the viruses recovered from the exposed animals (Table 2, Table S5) suggests that transmission of H9N2 viruses in ferrets may be independent of these changes, although such changes in PB2 could further increase their virulence and transmission, as was seen with the H5N1 and H7N9 viruses [48]–[50], [52], [60].

In addition to the receptor-binding preference conferred by HA, internal gene combinations also play a determinative role in virus transmissibility in mammals [2]. Similar to other avian influenza viruses circulating in poultry in Southern China [7], [61]–[63], the H9N2 viruses formed multiple genotypes. The DK/ZJ/C1036/09-like internal gene combination was detected in the H9N2 viruses with different groups of HA and NA genes that were isolated between 2009 and 2013 in nine of the 12 provinces investigated, and was also detected in the H7N9 and H10N8 viruses that have infected humans [12], [40] (Figure 1B), suggesting that this predominant internal gene combination is more stable and compatible with different surface genes. This internal gene combination also functions in transmission because it was present in all six of the transmissible viruses. Therefore, the H9N2 viruses pose a threat to human health not only because they will likely cause new influenza pandemic, but also because they can transfer different subtypes of influenza viruses from avian species to humans.

## Materials and Methods

### Ethics statements

This study was carried out in strict accordance with the recommendations in the Guide for the Care and Use of Laboratory Animals of the Ministry of Science and Technology of the People's Republic of China. The protocols for animal studies were approved by the Committee on the Ethics of Animal Experiments of the Harbin Veterinary Research Institute (HVRI) of the Chinese Academy of Agricultural Sciences (CAAS) (approval numbers BRDW-XBS–12 for mice and BRDW-XD–12 for ferrets).

### Facility

All experiments with live H9N2 viruses were conducted within the enhanced animal biosafety level 2+ (ABSL2+) facility in the HVRI of the CAAS. The animal isolators in the facility are hyper-filtered. The researchers who work with mice and ferrets wear N95 masks and disposable overalls; they shower on exiting the facility.

### Viruses and cells

The H9N2 viruses used in this study were isolated from poultry in different regions of China between 2009 and 2013. Virus stocks were grown in specific pathogen-free (SPF) chicken eggs. Madin-Darby canine kidney (MDCK) cells used for virus titration were cultured in DMEM (CORNING, Cellgro) medium with 4% fetal bovine serum (FBS). 293T cells were cultured in DMEM medium with 10% FBS. All cells were incubated at 37°C with 5% CO_2_.

### Genetic and phylogenetic analyses

Viral gene amplification and sequencing was carried out as described previously [18], [61]. Sequence data were compiled with the SEQMAN program (DNASTAR, Madison, WI), and phylogenetic analyses were carried out with the PHYLIP program of MEGA 5.0 software using the neighbor-joining algorithm. Bootstrap values of 1,000 were used, and 95% sequence identity cutoffs were used to categorize each gene segment in the phylogenetic trees.

### Receptor-binding analysis

Receptor specificity was analyzed by use of a solid-phase direct binding assay as described previously with modified using two different glycopolymers: α-2, 3-siaylglycopolymer [Neu5Acα2-3Galβ1-4GlcNAcβ1-pAP (para-aminophenyl)-alpha-polyglutamic acid (α-PGA)] and the α-2, 6-sialylglycopolymer [Neu5Acα2-6Galβ1-4GlcNAcβ1-pAP (para-aminophenyl)-alpha-polyglutamic acid (α-PGA)] [4], [7]. Briefly, viruses were grown in eggs, clarified by low-speed centrifugation, laid over a cushion of 30% sucrose in phosphate buffered saline (PBS), and ultracentrifuged at 28,000 r.p.m for 2 h at 4°C. Virus stocks were aliquoted and stored at −80°C until use. Virus concentrations were determined by using haemagglutination assays with 0.5% cRBCs. Microtitre plates (Nunc) were incubated with serial two-fold dilutions of sodium salts of sialyglycopolymers in PBS at 4°C for 30 min. Then the plates were exposed to UV light (254 nm) for 10 min. After the glycopolymer solution was removed, the plates were washed three times with PBS. Then 50 µl of virus suspensions diluted with PBST (PBS containing 0.1% Twen-20) was added to the wells and the plate was incubated at 4°C for 2 to 3 h. After being washed five times with 250 µl of PBST, the plates were fixed with 10% formalin in PBST for 30 min. After the plates were again washed five times with PBST, 50 µl of chicken antiserum against the CK/JS/C4258/12 (H9N2) virus diluted with PBST was added to the wells and incubated at 37°C for 1 h. After being washed for a further five times with PBST, the plates were incubated with a horseradish peroxidase (HRP)-conjugated goat-anti-chicken antibody (Sigma-Aldrich, St. Louis, MO, USA) for 1 h at 37°C.The plates were then washed again and incubated with *O*-phenylenediamine (Sigma-Aldrich, St. Louis, MO, USA) in PBS containing 0.01% H_2_O_2_ for 10 min at room temperature. The reaction was stopped with 0.05 ml of 0.5 M H_2_SO_4_. The optical density at 490 nm was determined in a microplate reader (BIO-RAD). Dose-response curves of virus binding to the glycopolymers were analyzed by using a single site binding algorithm and curve fitting by GraphPad Prism to determine the association constant values (K_a_). Each value presented is the mean ± SD of three experiments, which were each performed in triplicate.

### Site-directed mutagenesis and virus generation

The HA and NA segments of CK/GX/9/99 were inserted into the bidirectional transcription vector pBD as described previously [49]. The QuikChange Lighting Site-Directed Mutagenesis Kit (Stratagene, http://www.agilent.com) was used to create specific mutations in the HA gene by using the following primers: forward: 5′AGCATGAGATGGTTGATTCAA AAGGACAAC, reverse: 5′AGCGTTGTCCTTTTGAATCAACCATCTCAT (for HAT155I mutation); and forward: 5′ TTCATGTGGGGCATACATCACCCACCCACC, reverse: 5′TCGGTGGGTGGGTGA TGTATGCCCCACATG (for HAN183H mutation). The HA (with or without the mutations) and NA genes of the CK/GX/9/99 virus and the six internal genes of the PR8 virus were used to generate the viruses as previously described [49]. All HA and NA genes of the constructs and rescued viruses were completely sequenced to ensure the absence of unwanted mutations. The gene sequences of the CK/GX/9/99 virus used in this study have been reported previously [18].

### Mouse experiments

Groups of six-week-old female BALB/c mice (Beijing Vital River Laboratories, Beijing, China) were anesthetized with CO_2_ and inoculated intranasally (i.n.) with 10^6.0^ EID_50_ of test viruses in a volume of 50 µl. Three mice were euthanized on day 3 p.i., and the nasal turbinates, lungs, kidneys, spleens, and brains were collected for virus titration in MDCK cells. The remaining five mice in each group were monitored daily for 14 days for weight loss and survival.

### Ferret studies

Four-month-old female ferrets (Wuxi Cay Ferret Farm, Jiangsu, China) that were serologically negative for influenza viruses were used in these studies. The animals were anesthetized via intramuscular injection of ketamine (20 mg/kg) and xylazine (1 mg/kg). To examine virus replication, groups of two ferrets were anesthetized and inoculated i.n. with 10^6.0^ EID_50_ of test viruses in a 500 µl volume (250 µl per nostril). The ferrets were euthanized on day 4 p.i. and the nasal turbinates, tonsils, trachea, lung, spleen, kidneys, liver, and brain were collected for virus titration in MDCK cells. The lung tissue was also collected for histologic study as described previously [64].

For the respiratory droplet transmission studies, groups of three ferrets were inoculated i.n. with 10^6.0^ EID_50_ of test virus and housed in specially designed cages inside an isolator as described previously [40]. Twenty-four hours later, three naïve animals were placed in an adjacent cage. Nasal washes were collected at 2-day intervals, beginning on day 2 p.i. (1 day post-exposure) and titrated in MDCK cells. The ambient conditions for these studies were set at 20–22°C and 30%–40% relative humidity. The airflow in the isolator was horizontal with a speed of 0.1 m/s; the airflow direction was from the inoculated animals to the exposed animals.

### Deep sequencing

Viral RNA was extracted and converted to cDNA by use primer 5′AGC RAA AGC AGG. Specific amplification of a 1000-nucleotide PB2 fragment covering codons 627 to 701 was applied by use a pair of specific primers (Forward: 5′GCAACRGCTATYYTRAGGAAAGC; Reverse 5′ AGTAGAAACAAGGTCGTTTTTAAA). PCR fragments of each virus were pooled in equal concentrations, and libraries were created for each virus by using the Ion Xpress Plus Fragment Library Kit (Life Technologies). Sequencing runs were performed by using the Ion Torrent personal genome machine (PGM, Life Technologies). Sequence reads were sorted by the Ion Xpress Barcode Adaptors 1–18 (Life Technologies). Reads were aligned to the PB2 reference sequence of each virus by using CLC Genomics Workbench 5.0.1. The threshold for mutation detection was manually set at 1%.

The genome sequences of the 35 viruses reported in this study are available in GenBank with the access numbers of KM113042 – KM113321.

## Supporting Information

Figure S1 **Geographic location of the H9N2 viruses analyzed in this study.**
(PDF)

Figure S2 **Phylogenetic analyses of the seven genes of H9N2 viruses.** Phylogenetic analyses of the seven genes of the avian H9N2 influenza viruses isolated between 2009 and 2013. Trees were generated by using the neighbor-joining method (N-J Method) with the MEGA 5.0 program, and the tree topology was evaluated by means of 1000 bootstrap analyses. The trees of NA (A), PB2 (B), NP (E), M (F), and NS (G) were rooted to A/Equine/Prague/1/56(H7N7), the PB1 (D) and PA (E) trees were rooted to A/Equine/London/1416/73(H7N7). The regions of the nucleotide sequences used for the phylogenetic analysis were: NA, 87 to 1263; PB2, 185 to 2290; PB1, 31 to 2233; PA, 25 to 2129; NP, 35 to 1457; M, 56 to 960; and NS, 75 to 800. Sequences of viruses with names in black were downloaded from available databases; viruses with names in blue were sequenced in this study. Abbreviations are as follows: Br, Brambling; CAn, Canine; CK, Chicken; DK, Duck; Env, Environment; GS, Goose; Pf, Peregrine falcon; Pg, Pigeon; QA, Quail; SW, Swine; Ty, Turkey; AH, Anhui; BJ, Beijing; CA, California; CS, Chang Sha; CQ, Chongqing; DH, Donghu; FJ, Fujian; GD, Guangdong; GX, Guangxi; GZ, Guangzhou; HB, Hebei; HZ, Hangzhou; HN, Henan; HK, Hong Kong; HuB, Hubei; HuN, Hunan; JL, Jilin; JS, Jiangsu; SD, Shandong; SH, Shanghai; TZ, Taizhou; WI, Wisconsin; XJ, Xinjiang; XZ, Xuzhou; YZ, Yangzhou; YN, Yunnan; ZJ, Zhejiang.
(PDF)

Figure S3 **Characterization of the receptor-binding properties of H9N2 viruses.** The binding of the viruses to two different biotinylated glycans (α-2, 3 glycan, blue; α-2, 6 glycan, pink) was tested. The data shown are the means of three repeats; the error bars indicate the standard deviations.
(PDF)

Figure S4 **Histological lesions caused by H9N2 viruses in the lungs of ferrets.** Ferrets were euthanized on day 4 p.i. with 10^6^EID_50_ of test virus, and the lungs were collected for pathological study. The lungs of DK/ZJ/C2046/12 virus-inoculated animal showed only mild histopathological changes (H&E staining,) (**A**), whereas the lungs of CK/HuN/C4136/10 (**B**), CK/CQ/C1258/11 (**C**), CK/HuB/C4196/09 (**D**), CK/ZJ/SC324/13 (**E**), and CK/SH/SC197/13 (**F**) virus-inoculated ferrets showed severe pathological lesions (H&E staining). Viral antigen was detected in the epithelial cells of bronchus and alveoli by means of immunohistochemical (IHC) staining (**G**, from the lung samples of a ferret inoculated with CK/GX/C1435/12 virus; H, from the lung samples of a ferret inoculated with CK/SH/SC197/13 virus). Images A–F were taken at ×100 magnification; images **G** and **H** were taken at ×400 and ×200 magnification, respectively.
(PDF)

Table S1 **Mutations detected in the H9N2 viruses that contribute to the increased binding to human-type receptors, transmission, replication, and virulence in mammals, as well as to resistance to amantadine and rimantadine.**
(PDF)

Table S2 **Virus binding affinity to sialylglycopolymers.**
(PDF)

Table S3 **Virus selection scenarios for the ferret study.**
(PDF)

Table S4 **The body temperature increase and body weight loss of each of the ferrets inoculated with or exposed to the different H9N2 influenza viruses.**
(PDF)

Table S5 **Assessment of the presence of the 627K and 701N mutations in the PB2 segment of stock viruses and viruses recovered from the nasal washes of H9N2 influenza virus-infected or -exposed ferrets by use of deep sequencing.**
(PDF)
