# Correction: Genetics, Receptor Binding Property, and Transmissibility in Mammals of Naturally Isolated H9N2 Avian Influenza Viruses

**DOI:** 10.1371/journal.ppat.1008284

**Published:** 2020-01-08

**Authors:** Xuyong Li, Jianzhong Shi, Jing Guo, Guohua Deng, Qianyi Zhang, Jinliang Wang, Xijun He, Kaicheng Wang, Jiming Chen, Yuanyuan Li, Jun Fan, Huiui Kong, Chunyang Gu, Yuantao Guan, Yasuo Suzuki, Yoshihiro Kawaoka, Liling Liu, Yongping Jiang, Guobin Tian, Yanbing Li, Zhigao Bu, Hualan Chen

There is an error in Fig 3. The genotype number of DK/ZJ/C1036/09 virus and the maximum body weight change of mice infected with this virus are missing. Please see the correct Fig 3 here.

**Fig 3:**
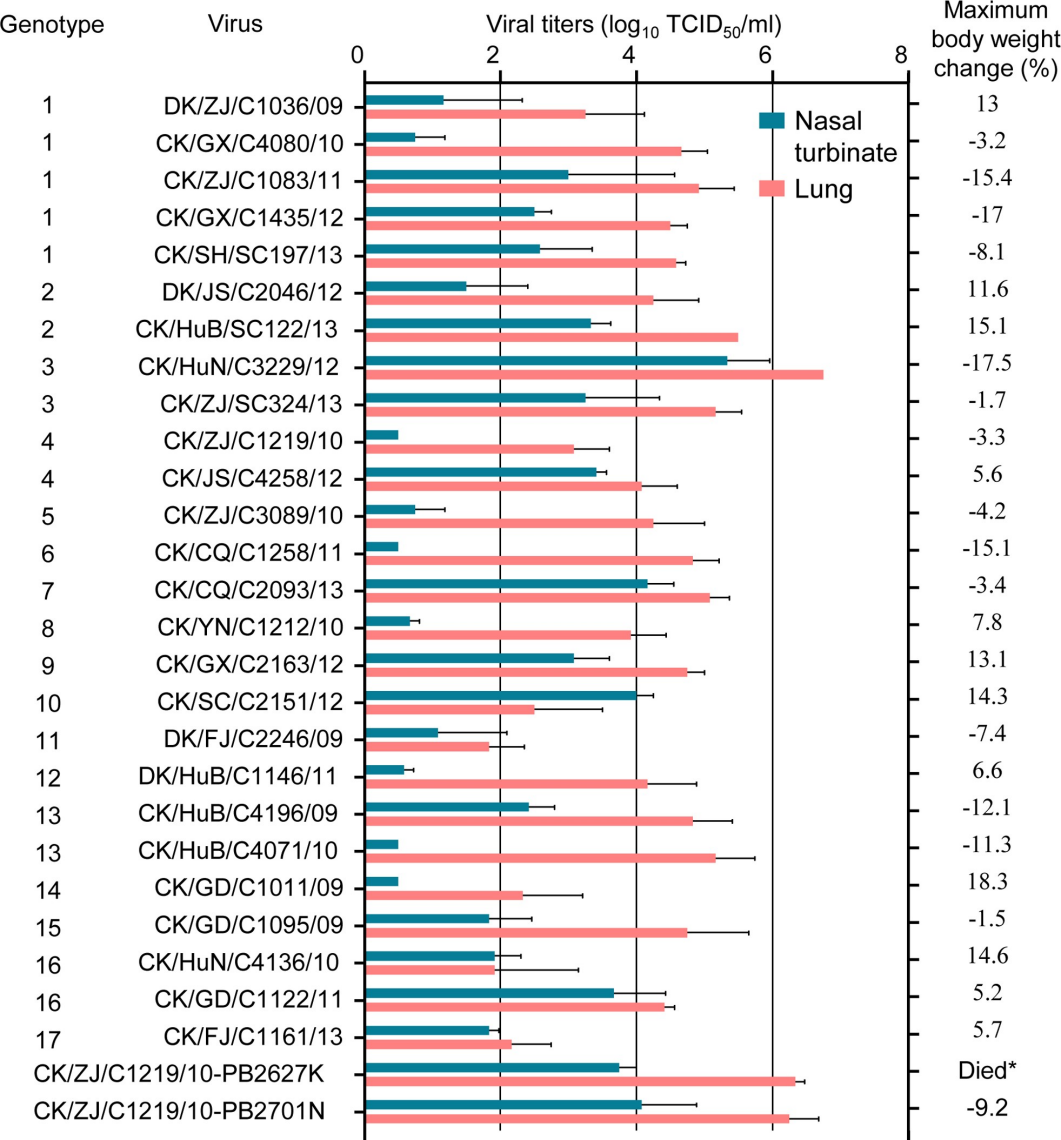
Replication and virulence of H9N2 viruses in mice. Virus titers in organs of mice on day 3 p.i. with 10^6^ EID_50_ of test virus. Data shown are the mean titers from three mice; the error bars indicate the standard deviations. *, mice inoculated with CK/ZJ/C1219/10-PB2/627K virus died before day 8 p.i. The dashed line indicates the lower limit of detection.
