# Supplementary figures and images for: Genetics, Receptor Binding Property, and Transmissibility in Mammals of Naturally Isolated H9N2 Avian Influenza Viruses
Source: PLoS Pathog. 2014 Nov 20;10(11):e1004508. doi: 10.1371/journal.ppat.1004508 (PMC4239090; doi:10.1371/journal.ppat.1004508)

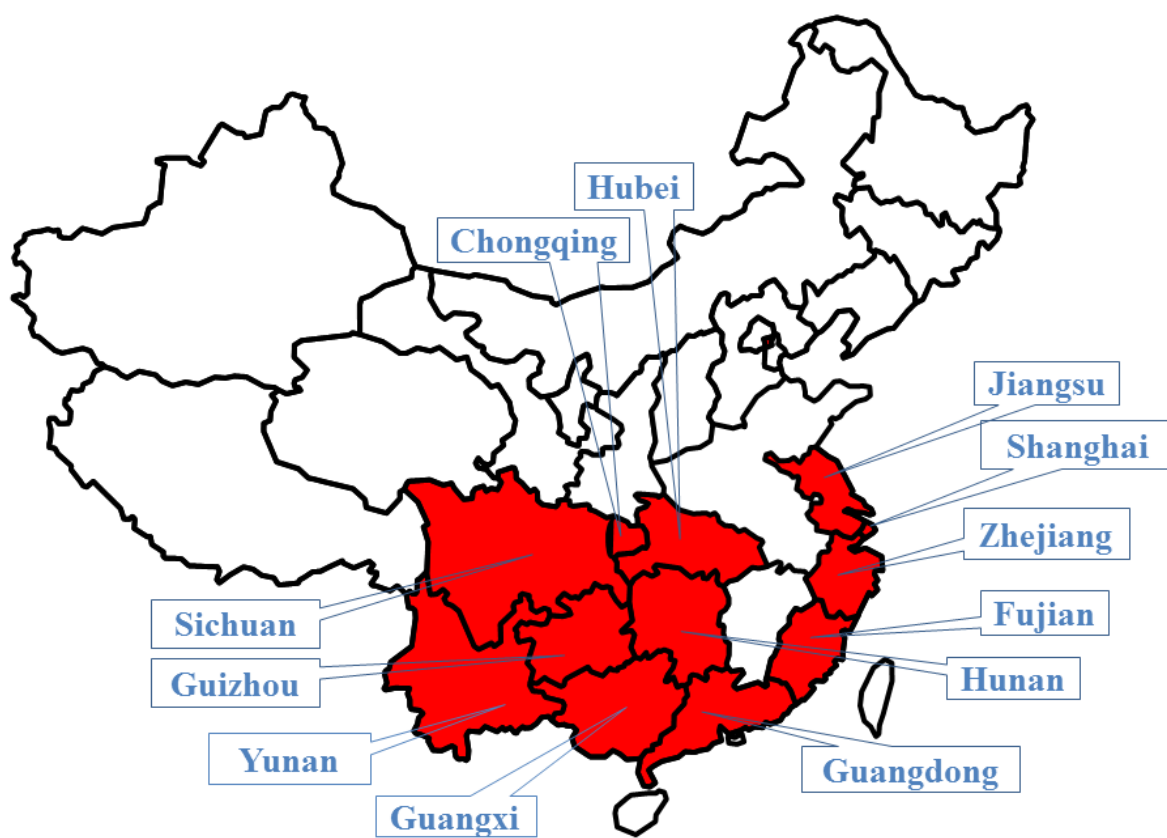

**Figure S1. Geographic location of the H9N2 viruses analyzed in this study.**

Supplement: Figure S1 — Geographic location of the H9N2 viruses analyzed in this study. (PDF) [file ppat.1004508.s001.pdf]
