# Supplementary material for: Genetics, Receptor Binding Property, and Transmissibility in Mammals of Naturally Isolated H9N2 Avian Influenza Viruses
Source: PLoS Pathog. 2014 Nov 20;10(11):e1004508. doi: 10.1371/journal.ppat.1004508 (PMC4239090; doi:10.1371/journal.ppat.1004508)
Supplement: Figure S2 — Phylogenetic analyses of the seven genes of H9N2 viruses. Phylogenetic analyses of the seven genes of the avian H9N2 influenza viruses isolated between 2009 and 2013. Trees were generated by using the neighbor-joining method (N-J Method) with the MEGA 5.0 program, and the tree topology was evaluated by means of 1000 bootstrap analyses. The trees of NA (A), PB2 (B), NP (E), M (F), and NS (G) were rooted to A/Equine/Prague/1/56(H7N7), the PB1 (D) and PA (E) trees were rooted to A/Equine/London/1416/73(H7N7). The regions of the nucleotide sequences used for the phylogenetic analysis were: NA, 87 to 1263; PB2, 185 to 2290; PB1, 31 to 2233; PA, 25 to 2129; NP, 35 to 1457; M, 56 to 960; and NS, 75 to 800. Sequences of viruses with names in black were downloaded from available databases; viruses with names in blue were sequenced in this study. Abbreviations are as follows: Br, Brambling; CAn, Canine; CK, Chicken; DK, Duck; Env, Environment; GS, Goose; Pf, Peregrine falcon; Pg, Pigeon; QA, Quail; SW, Swine; Ty, Turkey; AH, Anhui; BJ, Beijing; CA, California; CS, Chang Sha; CQ, Chongqing; DH, Donghu; FJ, Fujian; GD, Guangdong; GX, Guangxi; GZ, Guangzhou; HB, Hebei; HZ, Hangzhou; HN, Henan; HK, Hong Kong; HuB, Hubei; HuN, Hunan; JL, Jilin; JS, Jiangsu; SD, Shandong; SH, Shanghai; TZ, Taizhou; WI, Wisconsin; XJ, Xinjiang; XZ, Xuzhou; YZ, Yangzhou; YN, Yunnan; ZJ, Zhejiang. (PDF) [file ppat.1004508.s002.pdf]

Fig.S2 A NA

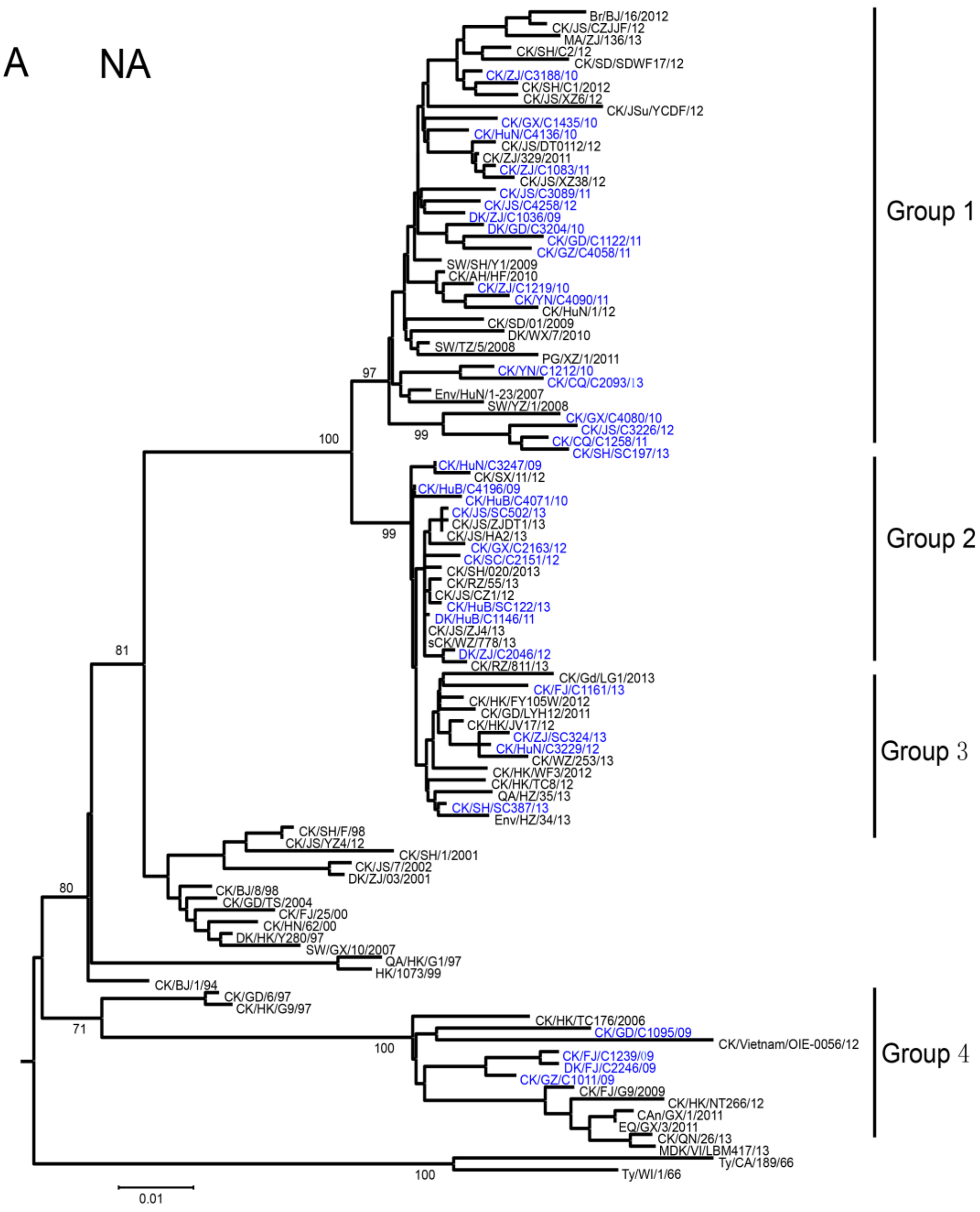
Br/BJ/16/2012
CK/JS/CZJJF/12
MA/ZJ/136/13
CK/SH/C2/12
CK/SD/SDWF17/12
CK/ZJ/C3188/10
CK/SH/C1/2012
CK/JS/XZ6/12
CK/JSu/YCDF/12
CK/GX/C1435/10
CK/HuN/C4136/10
CK/JS/DT0112/12
CK/ZJ/329/2011
CK/ZJ/C1083/11
CK/JS/XZ38/12
CK/JS/C3089/11
CK/JS/C4258/12
DK/ZJ/C1036/09
DK/GD/C3204/10
CK/GD/C1122/11
CK/GZ/C4058/11
SW/SH/Y1/2009
CK/AH/HF/2010
CK/ZJ/C1219/10
CK/YN/C4090/11
CK/HuN/1/12
CK/SD/01/2009
DK/WX/7/2010
SW/TZ/5/2008
PG/XZ/1/2011
97
CK/YN/C1212/10
CK/CQ/C2093/13
Env/HuN/1-23/2007
SW/YZ/1/2008
CK/GX/C4080/10
CK/JS/C3226/12
99
CK/CQ/C1258/11
100
CK/SH/SC197/13
CK/HuN/C3247/09
CK/SX/11/12
CK/HuB/C4196/09
CK/HuB/C4071/10
CK/JS/SC502/13
CK/JS/ZJDT1/13
99
CK/JS/HA2/13
CK/GX/C2163/12
CK/SC/C2151/12
CK/SH/020/2013
CK/RZ/55/13
CK/JS/CZ1/12
CK/HuB/SC122/13
DK/HuB/C1146/11
CK/JS/ZJ4/13
sCK/WZ/778/13
81
DK/ZJ/C2046/12
CK/RZ/811/13
CK/Gd/LG1/2013
CK/FJ/C1161/13
CK/HK/FY105W/2012
CK/GD/LYH12/2011
CK/HK/JV17/12
CK/ZJ/SC324/13
CK/HuN/C3229/12
CK/WZ/253/13
CK/HK/WF3/2012
CK/HK/TC8/12
QA/HZ/35/13
CK/SH/SC387/13
Env/HZ/34/13
Group 1
Group 2
Group 3
CK/SH/F/98
CK/JS/YZ4/12
CK/SH/1/2001
CK/JS/7/2002
DK/ZJ/03/2001
80
CK/BJ/8/98
CK/GD/TS/2004
CK/FJ/25/00
CK/HN/62/00
DK/HK/Y280/97
SW/GX/10/2007
QA/HK/G1/97
HK/1073/99
CK/BJ/1/94
CK/GD/6/97
CK/HK/G9/97
71
CK/HK/TC176/2006
CK/GD/C1095/09
CK/Vietnam/OIE-0056/12
100
CK/FJ/C1239/09
DK/FJ/C2246/09
CK/GZ/C1011/09
CK/FJ/G9/2009
CK/HK/NT266/12
CAn/GX/1/2011
EQ/GX/3/2011
CK/QN/26/13
MDK/VI/LBM417/13
Ty/CA/189/66
100
Ty/WI/1/66
Group 4
0.01

Fig.S2 B PB2

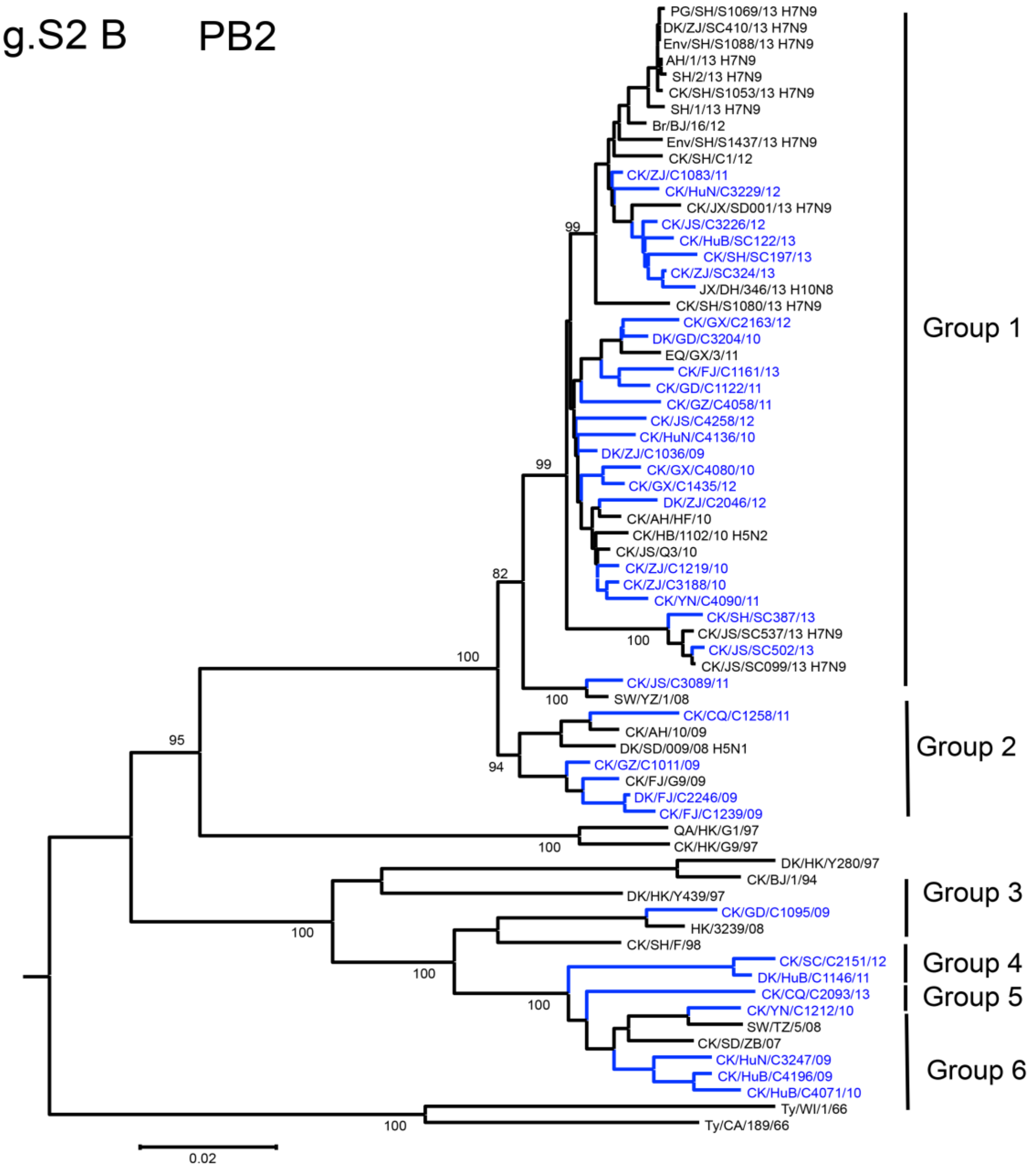
PG/SH/S1069/13 H7N9
DK/ZJ/SC410/13 H7N9
Env/SH/S1088/13 H7N9
AH/1/13 H7N9
SH/2/13 H7N9
CK/SH/S1053/13 H7N9
SH/1/13 H7N9
Br/BJ/16/12
Env/SH/S1437/13 H7N9
CK/SH/C1/12
CK/ZJ/C1083/11
CK/HuN/C3229/12
CK/JX/SD001/13 H7N9
CK/JS/C3226/12
CK/HuB/SC122/13
CK/SH/SC197/13
CK/ZJ/SC324/13
JX/DH/346/13 H10N8
CK/SH/S1080/13 H7N9
CK/GX/C2163/12
DK/GD/C3204/10
EQ/GX/3/11
CK/FJ/C1161/13
CK/GD/C1122/11
CK/GZ/C4058/11
CK/JS/C4258/12
CK/HuN/C4136/10
DK/ZJ/C1036/09
CK/GX/C4080/10
CK/GX/C1435/12
DK/ZJ/C2046/12
CK/AH/HF/10
CK/HB/1102/10 H5N2
CK/JS/Q3/10
CK/ZJ/C1219/10
CK/ZJ/C3188/10
CK/YN/C4090/11
CK/SH/SC387/13
CK/JS/SC537/13 H7N9
CK/JS/SC502/13
CK/JS/SC099/13 H7N9
CK/JS/C3089/11
SW/YZ/1/08
CK/CQ/C1258/11
CK/AH/10/09
DK/SD/009/08 H5N1
CK/GZ/C1011/09
CK/FJ/G9/09
DK/FJ/C2246/09
CK/FJ/C1239/09
QA/HK/G1/97
CK/HK/G9/97
DK/HK/Y280/97
CK/BJ/1/94
DK/HK/Y439/97
CK/GD/C1095/09
HK/3239/08
CK/SH/F/98
CK/SC/C2151/12
DK/HuB/C1146/11
CK/CQ/C2093/13
CK/YN/C1212/10
SW/TZ/5/08
CK/SD/ZB/07
CK/HuN/C3247/09
CK/HuB/C4196/09
CK/HuB/C4071/10
Ty/WI/1/66
Ty/CA/189/66
99
99
82
100
100
100
95
94
100
100
100
100
100
100
0.02
Group 1
Group 2
Group 3
Group 4
Group 5
Group 6

Fig.S2 C PB1

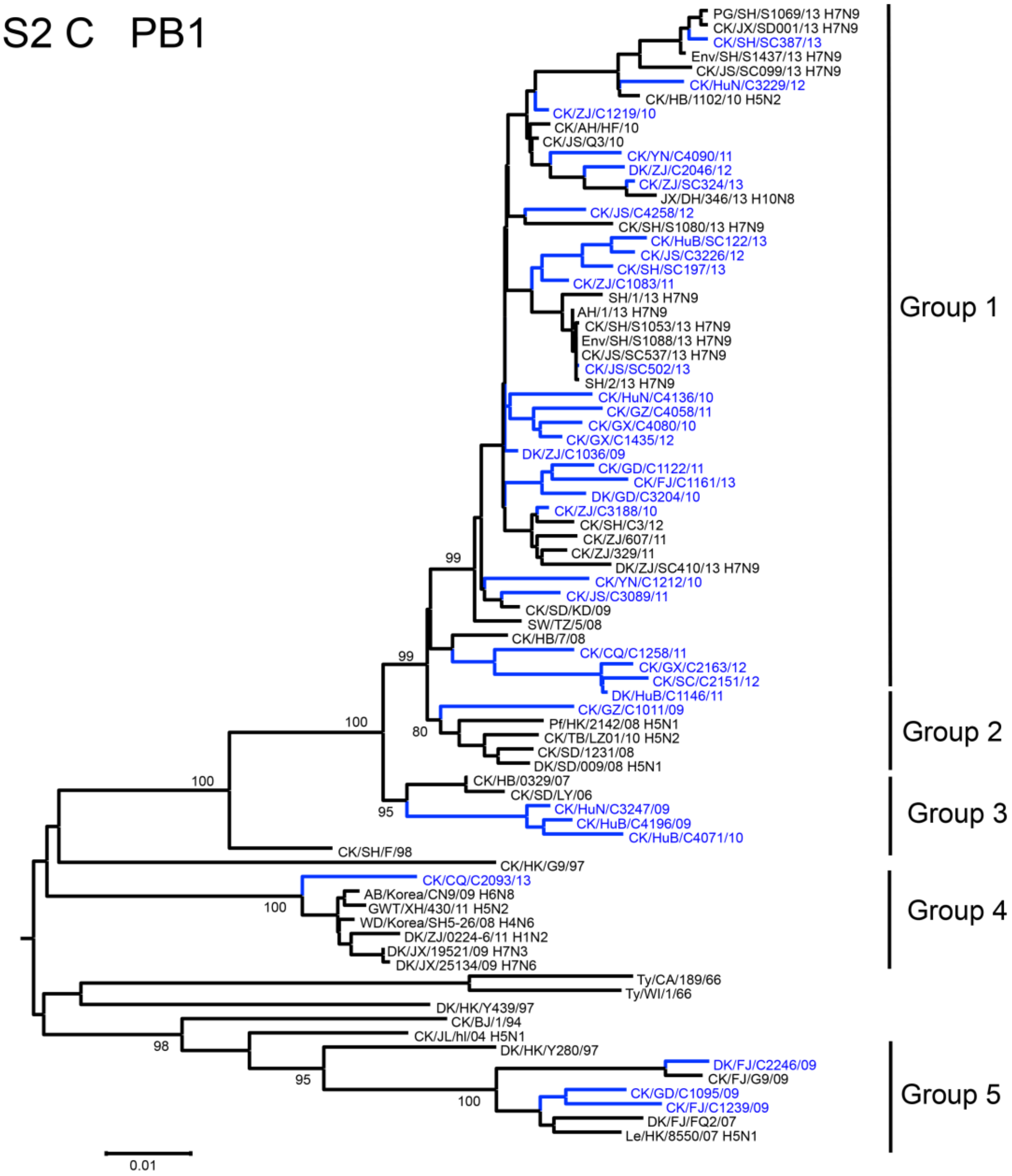
PG/SH/S1069/13 H7N9
CK/JX/SD001/13 H7N9
CK/SH/SC387/13
Env/SH/S1437/13 H7N9
CK/JS/SC099/13 H7N9
CK/HuN/C3229/12
CK/HB/1102/10 H5N2
CK/ZJ/C1219/10
CK/AH/HF/10
CK/JS/Q3/10
CK/YN/C4090/11
DK/ZJ/C2046/12
CK/ZJ/SC324/13
JX/DH/346/13 H10N8
CK/JS/C4258/12
CK/SH/S1080/13 H7N9
CK/HuB/SC122/13
CK/JS/C3226/12
CK/SH/SC197/13
CK/ZJ/C1083/11
SH/1/13 H7N9
AH/1/13 H7N9
CK/SH/S1053/13 H7N9
Env/SH/S1088/13 H7N9
CK/JS/SC537/13 H7N9
CK/JS/SC502/13
SH/2/13 H7N9
CK/HuN/C4136/10
CK/GZ/C4058/11
CK/GX/C4080/10
CK/GX/C1435/12
DK/ZJ/C1036/09
CK/GD/C1122/11
CK/FJ/C1161/13
DK/GD/C3204/10
CK/ZJ/C3188/10
CK/SH/C3/12
CK/ZJ/607/11
CK/ZJ/329/11
DK/ZJ/SC410/13 H7N9
99
CK/YN/C1212/10
CK/JS/C3089/11
CK/SD/KD/09
SW/TZ/5/08
CK/HB/7/08
99
CK/CQ/C1258/11
CK/GX/C2163/12
CK/SC/C2151/12
DK/HuB/C1146/11
CK/GZ/C1011/09
100
80
Pf/HK/2142/08 H5N1
CK/TB/LZ01/10 H5N2
CK/SD/1231/08
DK/SD/009/08 H5N1
100
CK/HB/0329/07
CK/SD/LY/06
95
CK/HuN/C3247/09
CK/HuB/C4196/09
CK/HuB/C4071/10
CK/SH/F/98
CK/HK/G9/97
CK/CQ/C2093/13
AB/Korea/CN9/09 H6N8
100
GWT/XH/430/11 H5N2
WD/Korea/SH5-26/08 H4N6
DK/ZJ/0224-6/11 H1N2
DK/JX/19521/09 H7N3
DK/JX/25134/09 H7N6
Ty/CA/189/66
Ty/WI/1/66
DK/HK/Y439/97
CK/BJ/1/94
CK/JL/hl/04 H5N1
98
DK/HK/Y280/97
DK/FJ/C2246/09
95
CK/FJ/G9/09
100
CK/GD/C1095/09
CK/FJ/C1239/09
DK/FJ/FQ2/07
Le/HK/8550/07 H5N1
0.01
Group 1
Group 2
Group 3
Group 4
Group 5

Fig.S2 D PA

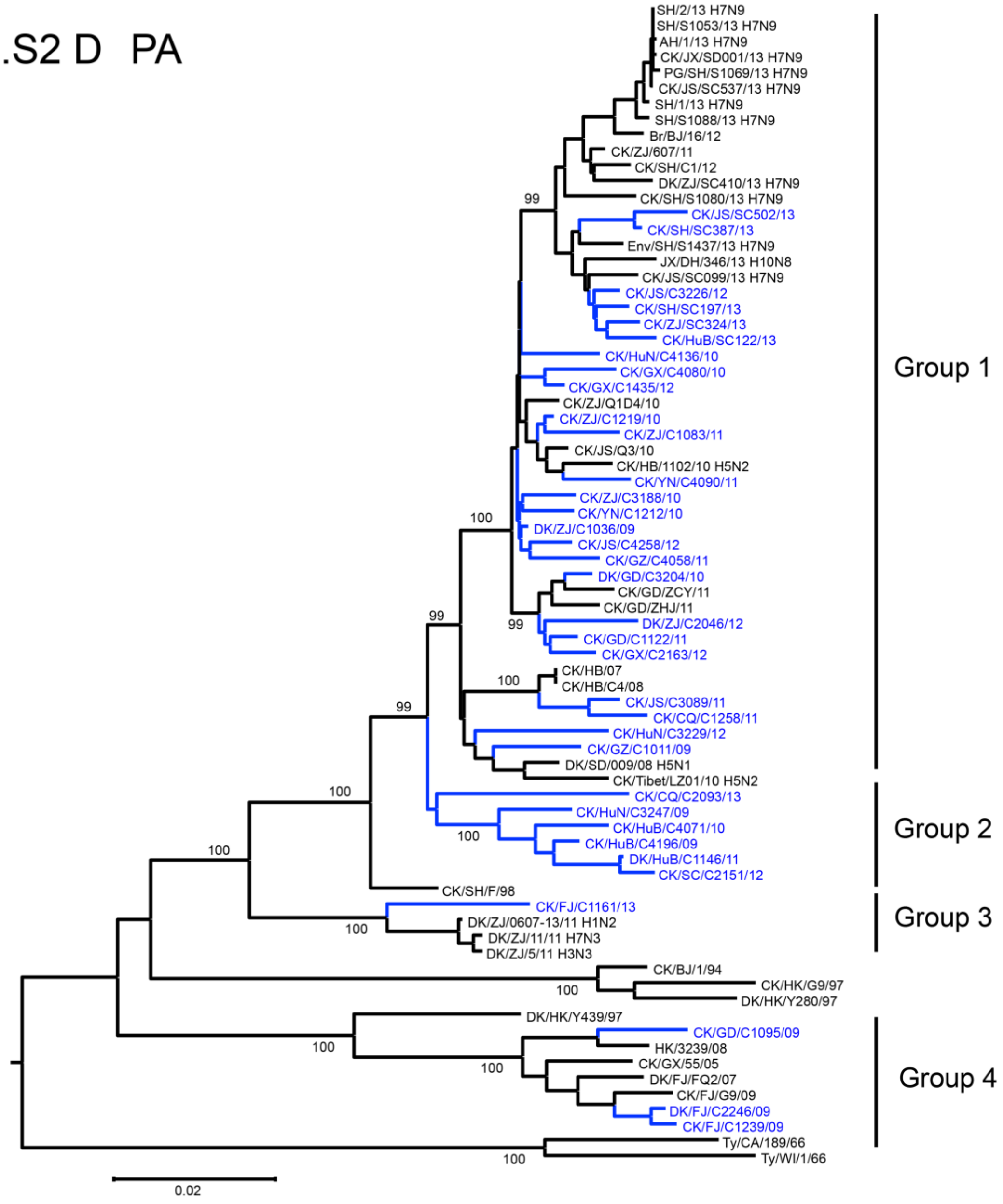
SH/2/13 H7N9
SH/S1053/13 H7N9
AH/1/13 H7N9
CK/JX/SD001/13 H7N9
PG/SH/S1069/13 H7N9
CK/JS/SC537/13 H7N9
SH/1/13 H7N9
SH/S1088/13 H7N9
Br/BJ/16/12
CK/ZJ/607/11
CK/SH/C1/12
DK/ZJ/SC410/13 H7N9
CK/SH/S1080/13 H7N9
CK/JS/SC502/13
CK/SH/SC387/13
Env/SH/S1437/13 H7N9
JX/DH/346/13 H10N8
CK/JS/SC099/13 H7N9
CK/JS/C3226/12
CK/SH/SC197/13
CK/ZJ/SC324/13
CK/HuB/SC122/13
CK/HuN/C4136/10
CK/GX/C4080/10
CK/GX/C1435/12
CK/ZJ/Q1D4/10
CK/ZJ/C1219/10
CK/ZJ/C1083/11
CK/JS/Q3/10
CK/HB/1102/10 H5N2
CK/YN/C4090/11
CK/ZJ/C3188/10
CK/YN/C1212/10
DK/ZJ/C1036/09
CK/JS/C4258/12
CK/GZ/C4058/11
DK/GD/C3204/10
CK/GD/ZCY/11
CK/GD/ZHJ/11
DK/ZJ/C2046/12
CK/GD/C1122/11
CK/GX/C2163/12
CK/HB/07
CK/HB/C4/08
CK/JS/C3089/11
CK/CQ/C1258/11
CK/HuN/C3229/12
CK/GZ/C1011/09
DK/SD/009/08 H5N1
CK/Tibet/LZ01/10 H5N2
CK/CQ/C2093/13
CK/HuN/C3247/09
CK/HuB/C4071/10
CK/HuB/C4196/09
DK/HuB/C1146/11
CK/SC/C2151/12
CK/SH/F/98
CK/FJ/C1161/13
DK/ZJ/0607-13/11 H1N2
DK/ZJ/11/11 H7N3
DK/ZJ/5/11 H3N3
CK/BJ/1/94
CK/HK/G9/97
DK/HK/Y280/97
DK/HK/Y439/97
CK/GD/C1095/09
HK/3239/08
CK/GX/55/05
DK/FJ/FQ2/07
CK/FJ/G9/09
DK/FJ/C2246/09
CK/FJ/C1239/09
Ty/CA/189/66
Ty/WI/1/66
99
100
99
99
99
100
100
100
100
100
100
100
100
100
100
0.02
Group 1
Group 2
Group 3
Group 4

Fig.S2 E   NP

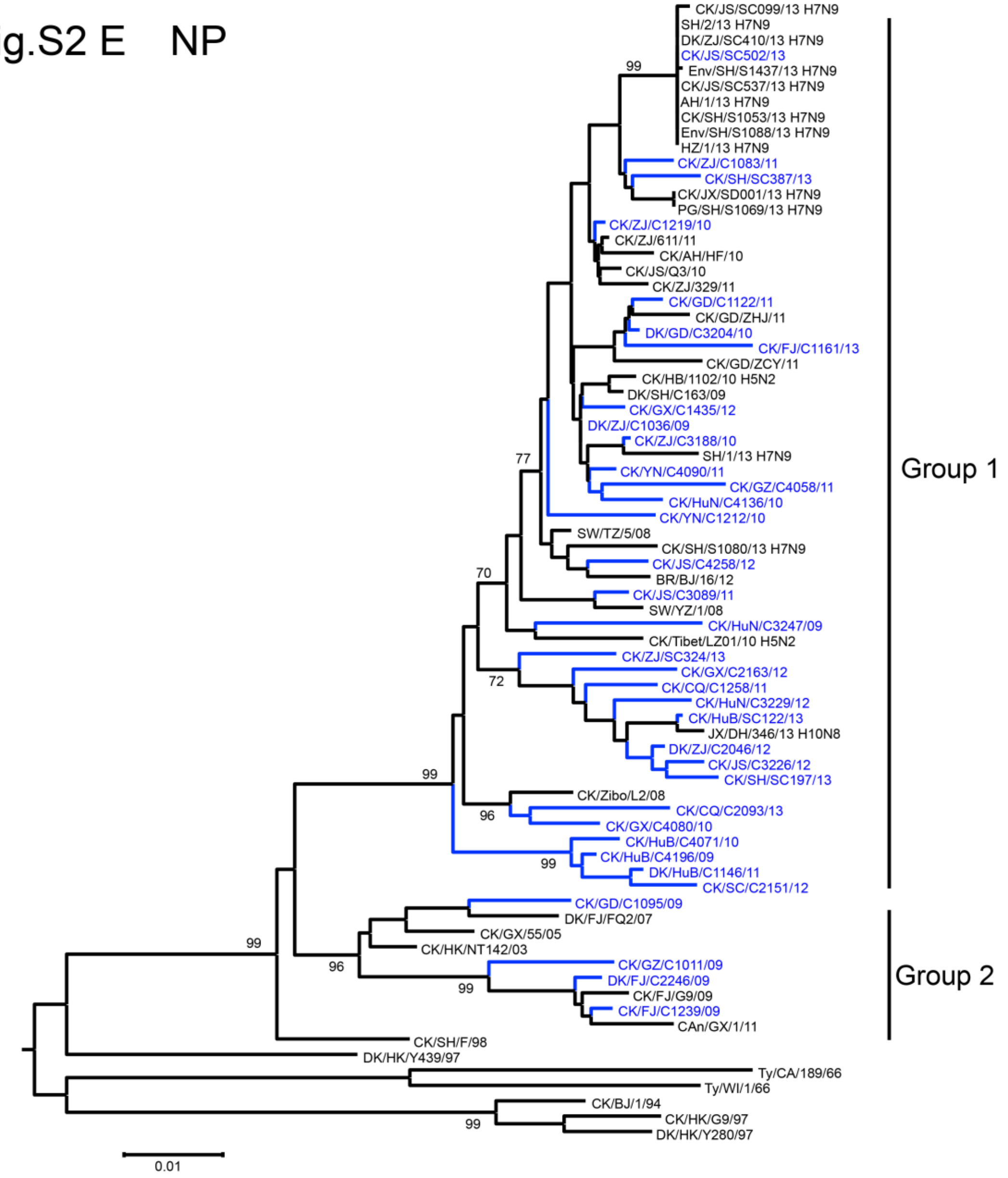
CK/JS/SC099/13 H7N9
SH/2/13 H7N9
DK/ZJ/SC410/13 H7N9
CK/JS/SC502/13
Env/SH/S1437/13 H7N9
CK/JS/SC537/13 H7N9
AH/1/13 H7N9
CK/SH/S1053/13 H7N9
Env/SH/S1088/13 H7N9
HZ/1/13 H7N9
CK/ZJ/C1083/11
CK/SH/SC387/13
CK/JX/SD001/13 H7N9
PG/SH/S1069/13 H7N9
CK/ZJ/C1219/10
CK/ZJ/611/11
CK/AH/HF/10
CK/JS/Q3/10
CK/ZJ/329/11
CK/GD/C1122/11
CK/GD/ZHJ/11
DK/GD/C3204/10
CK/FJ/C1161/13
CK/GD/ZCY/11
CK/HB/1102/10 H5N2
DK/SH/C163/09
CK/GX/C1435/12
DK/ZJ/C1036/09
CK/ZJ/C3188/10
SH/1/13 H7N9
CK/YN/C4090/11
CK/GZ/C4058/11
CK/HuN/C4136/10
CK/YN/C1212/10
SW/TZ/5/08
CK/SH/S1080/13 H7N9
CK/JS/C4258/12
BR/BJ/16/12
CK/JS/C3089/11
SW/YZ/1/08
CK/HuN/C3247/09
CK/Tibet/LZ01/10 H5N2
CK/ZJ/SC324/13
CK/GX/C2163/12
CK/CQ/C1258/11
CK/HuN/C3229/12
CK/HuB/SC122/13
JX/DH/346/13 H10N8
DK/ZJ/C2046/12
CK/JS/C3226/12
CK/SH/SC197/13
CK/Zibo/L2/08
CK/CQ/C2093/13
CK/GX/C4080/10
CK/HuB/C4071/10
CK/HuB/C4196/09
DK/HuB/C1146/11
CK/SC/C2151/12
CK/GD/C1095/09
DK/FJ/FQ2/07
CK/GX/55/05
CK/HK/NT142/03
CK/GZ/C1011/09
DK/FJ/C2246/09
CK/FJ/G9/09
CK/FJ/C1239/09
CAn/GX/1/11
CK/SH/F/98
DK/HK/Y439/97
Ty/CA/189/66
Ty/WI/1/66
CK/BJ/1/94
CK/HK/G9/97
DK/HK/Y280/97
99
77
70
72
99
96
99
99
96
99
99
Group 1
Group 2
0.01

Fig.S2 F M

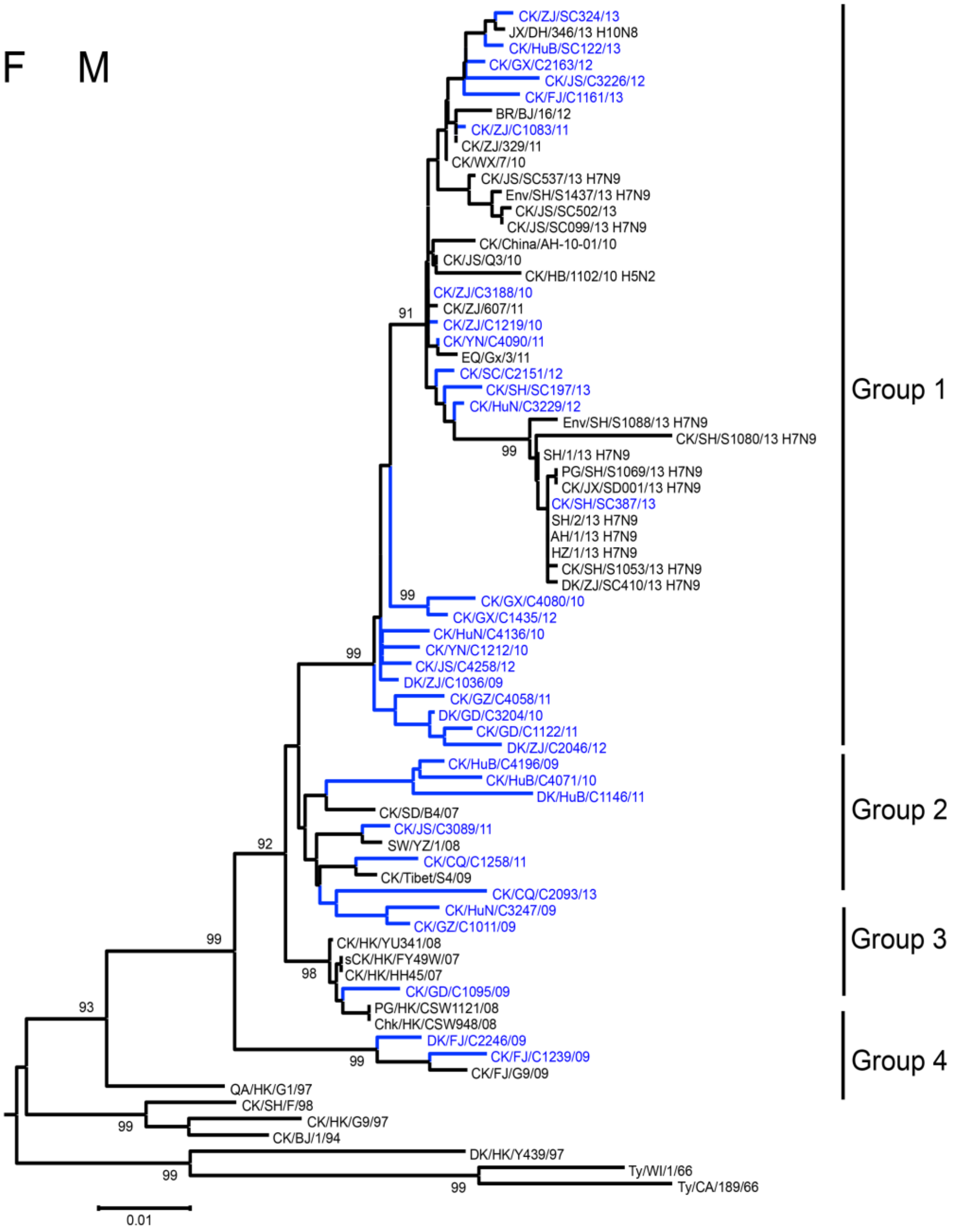
CK/ZJ/SC324/13
JX/DH/346/13 H10N8
CK/HuB/SC122/13
CK/GX/C2163/12
CK/JS/C3226/12
CK/FJ/C1161/13
BR/BJ/16/12
CK/ZJ/C1083/11
CK/ZJ/329/11
CK/WX/7/10
CK/JS/SC537/13 H7N9
Env/SH/S1437/13 H7N9
CK/JS/SC502/13
CK/JS/SC099/13 H7N9
CK/China/AH-10-01/10
CK/JS/Q3/10
CK/HB/1102/10 H5N2
CK/ZJ/C3188/10
91
CK/ZJ/607/11
CK/ZJ/C1219/10
CK/YN/C4090/11
EQ/Gx/3/11
CK/SC/C2151/12
CK/SH/SC197/13
CK/HuN/C3229/12
Env/SH/S1088/13 H7N9
CK/SH/S1080/13 H7N9
99
SH/1/13 H7N9
PG/SH/S1069/13 H7N9
CK/JX/SD001/13 H7N9
CK/SH/SC387/13
SH/2/13 H7N9
AH/1/13 H7N9
HZ/1/13 H7N9
CK/SH/S1053/13 H7N9
DK/ZJ/SC410/13 H7N9
99
CK/GX/C4080/10
CK/GX/C1435/12
CK/HuN/C4136/10
99
CK/YN/C1212/10
CK/JS/C4258/12
DK/ZJ/C1036/09
CK/GZ/C4058/11
DK/GD/C3204/10
CK/GD/C1122/11
DK/ZJ/C2046/12
CK/HuB/C4196/09
CK/HuB/C4071/10
DK/HuB/C1146/11
CK/SD/B4/07
CK/JS/C3089/11
92
SW/YZ/1/08
CK/CQ/C1258/11
CK/Tibet/S4/09
CK/CQ/C2093/13
CK/HuN/C3247/09
CK/GZ/C1011/09
99
CK/HK/YU341/08
sCK/HK/FY49W/07
98
CK/HK/HH45/07
CK/GD/C1095/09
PG/HK/CSW1121/08
93
Chk/HK/CSW948/08
DK/FJ/C2246/09
CK/FJ/C1239/09
99
CK/FJ/G9/09
QA/HK/G1/97
CK/SH/F/98
CK/HK/G9/97
99
CK/BJ/1/94
DK/HK/Y439/97
Ty/WI/1/66
99
99
Ty/CA/189/66
0.01
Group 1
Group 2
Group 3
Group 4

Fig.S2. G NS

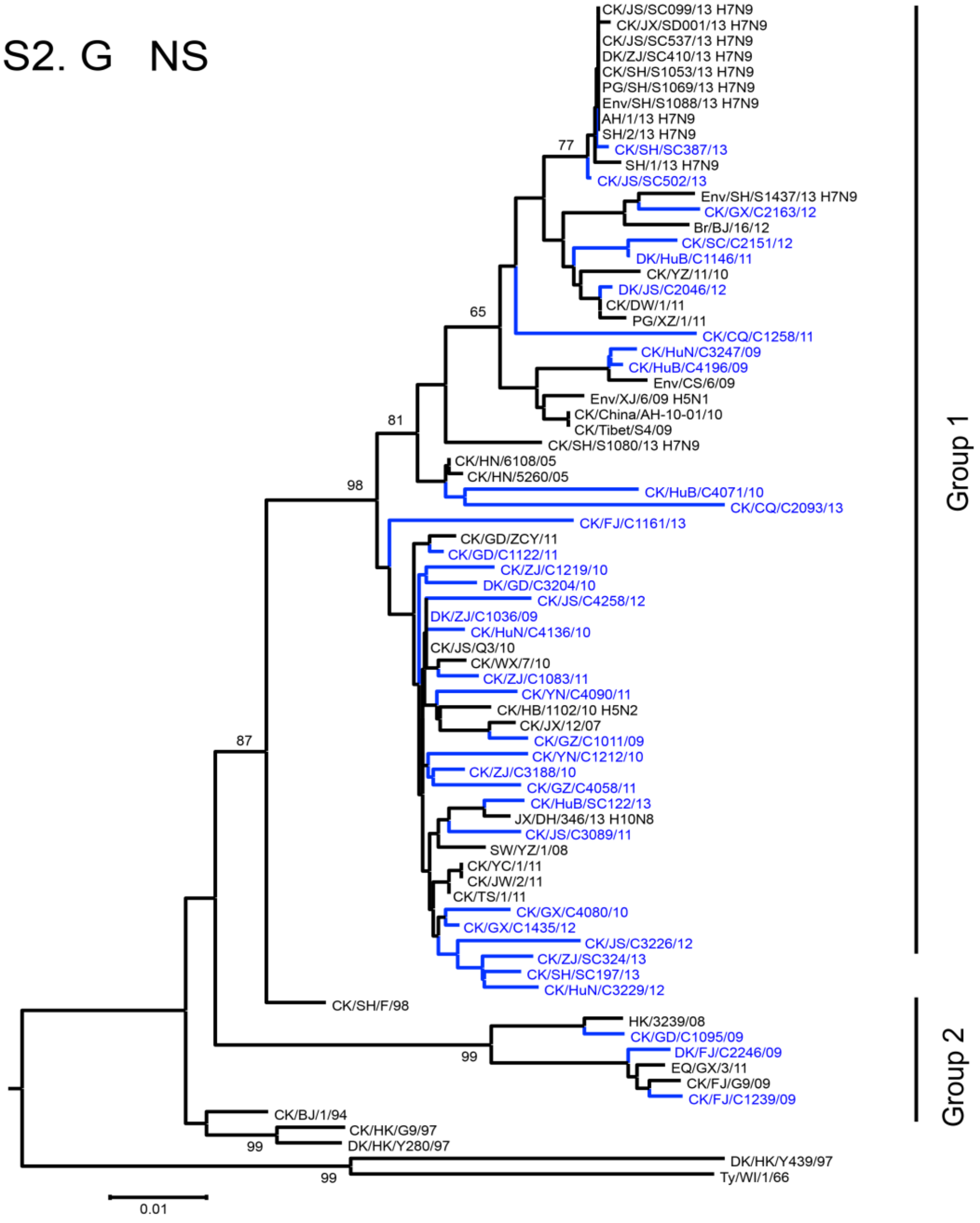
CK/JS/SC099/13 H7N9
CK/JX/SD001/13 H7N9
CK/JS/SC537/13 H7N9
DK/ZJ/SC410/13 H7N9
CK/SH/S1053/13 H7N9
PG/SH/S1069/13 H7N9
Env/SH/S1088/13 H7N9
AH/1/13 H7N9
SH/2/13 H7N9
77
CK/SH/SC387/13
SH/1/13 H7N9
CK/JS/SC502/13
Env/SH/S1437/13 H7N9
CK/GX/C2163/12
Br/BJ/16/12
CK/SC/C2151/12
DK/HuB/C1146/11
CK/YZ/11/10
DK/JS/C2046/12
CK/DW/1/11
PG/XZ/1/11
65
CK/CQ/C1258/11
CK/HuN/C3247/09
CK/HuB/C4196/09
Env/CS/6/09
Env/XJ/6/09 H5N1
CK/China/AH-10-01/10
CK/Tibet/S4/09
81
CK/SH/S1080/13 H7N9
CK/HN/6108/05
CK/HN/5260/05
98
CK/HuB/C4071/10
CK/CQ/C2093/13
CK/FJ/C1161/13
CK/GD/ZCY/11
CK/GD/C1122/11
CK/ZJ/C1219/10
DK/GD/C3204/10
CK/JS/C4258/12
DK/ZJ/C1036/09
CK/HuN/C4136/10
CK/JS/Q3/10
CK/WX/7/10
CK/ZJ/C1083/11
CK/YN/C4090/11
CK/HB/1102/10 H5N2
CK/JX/12/07
87
CK/GZ/C1011/09
CK/YN/C1212/10
CK/ZJ/C3188/10
CK/GZ/C4058/11
CK/HuB/SC122/13
JX/DH/346/13 H10N8
CK/JS/C3089/11
SW/YZ/1/08
CK/YC/1/11
CK/JW/2/11
CK/TS/1/11
CK/GX/C4080/10
CK/GX/C1435/12
CK/JS/C3226/12
CK/ZJ/SC324/13
CK/SH/SC197/13
CK/HuN/C3229/12
CK/SH/F/98
HK/3239/08
CK/GD/C1095/09
99
DK/FJ/C2246/09
EQ/GX/3/11
CK/FJ/G9/09
CK/FJ/C1239/09
CK/BJ/1/94
CK/HK/G9/97
99
DK/HK/Y280/97
DK/HK/Y439/97
99
Ty/WI/1/66
0.01
Group 1
Group 2

Figure S2. Phylogenetic analyses of the seven genes of H9N2 viruses. Phylogenetic analyses of the seven genes of the avian H9N2 influenza viruses isolated between 2009 and 2013.Trees were generated by using the neighbor-joining method (N-J Method) with the MEGA 5.0 program, and the tree topology was evaluated by means of 1000 bootstrap analyses. (**A**) NA gene tree. (**B**) PB2 gene tree. (**C**) PB1 gene tree. (**D**) PA gene tree. (**E**) NP gene tree. (**F**) M gene tree. (**G**) NS gene tree. The trees of NA, PB2, NP, M, and NS were rooted to A/Equine/Prague/1/56(H7N7), the PB1 and PA trees were rooted to A/Equine/London/1416/73(H7N7). The regions of the nucleotide sequences used for the phylogenetic analysis were: NA, 87 to 1263; PB2, 185 to 2290; PB1, 31 to 2233; PA, 25 to 2129; NP, 35 to 1457; M, 56 to 960; and NS, 75 to 800. Sequences of viruses with names in black were downloaded from available databases; viruses with names in blue were sequenced in this study. Abbreviations are as follows: Br, Brambling; CAn, Canine; CK, Chicken; DK, Duck; Env, Environment; GS, Goose; Pf, Peregrine falcon; Pg, Pigeon; QA, Quail ; SW, Swine; Ty , Turkey; AH, Anhui; BJ, Beijing; CA, California; CS, Chang Sha ; CQ, Chongqing ; DH, Donghu; FJ, Fujian; GD, Guangdong ; GX, Guangxi ; GZ, Guangzhou; HB, Hebei ; HZ, Hangzhou; HN, Henan; HK, Hong Kong ; HuB, Hubei ; HuN, Hunan; JL, Jilin; JS, Jiangsu ; SD, Shandong ; SH, Shanghai; TZ, Taizhou; WI, Wisconsin ; XJ, Xinjiang; XZ, Xuzhou; YZ, Yangzhou ; YN, Yunnan; ZJ, Zhejiang.
