# Supplementary material for: Genetics, Receptor Binding Property, and Transmissibility in Mammals of Naturally Isolated H9N2 Avian Influenza Viruses
Source: PLoS Pathog. 2014 Nov 20;10(11):e1004508. doi: 10.1371/journal.ppat.1004508 (PMC4239090; doi:10.1371/journal.ppat.1004508)
Supplement: Figure S3 — Characterization of the receptor-binding properties of H9N2 viruses. The binding of the viruses to two different biotinylated glycans (α-2, 3 glycan, blue; α-2, 6 glycan, pink) was tested. The data shown are the means of three repeats; the error bars indicate the standard deviations. (PDF) [file ppat.1004508.s003.pdf]

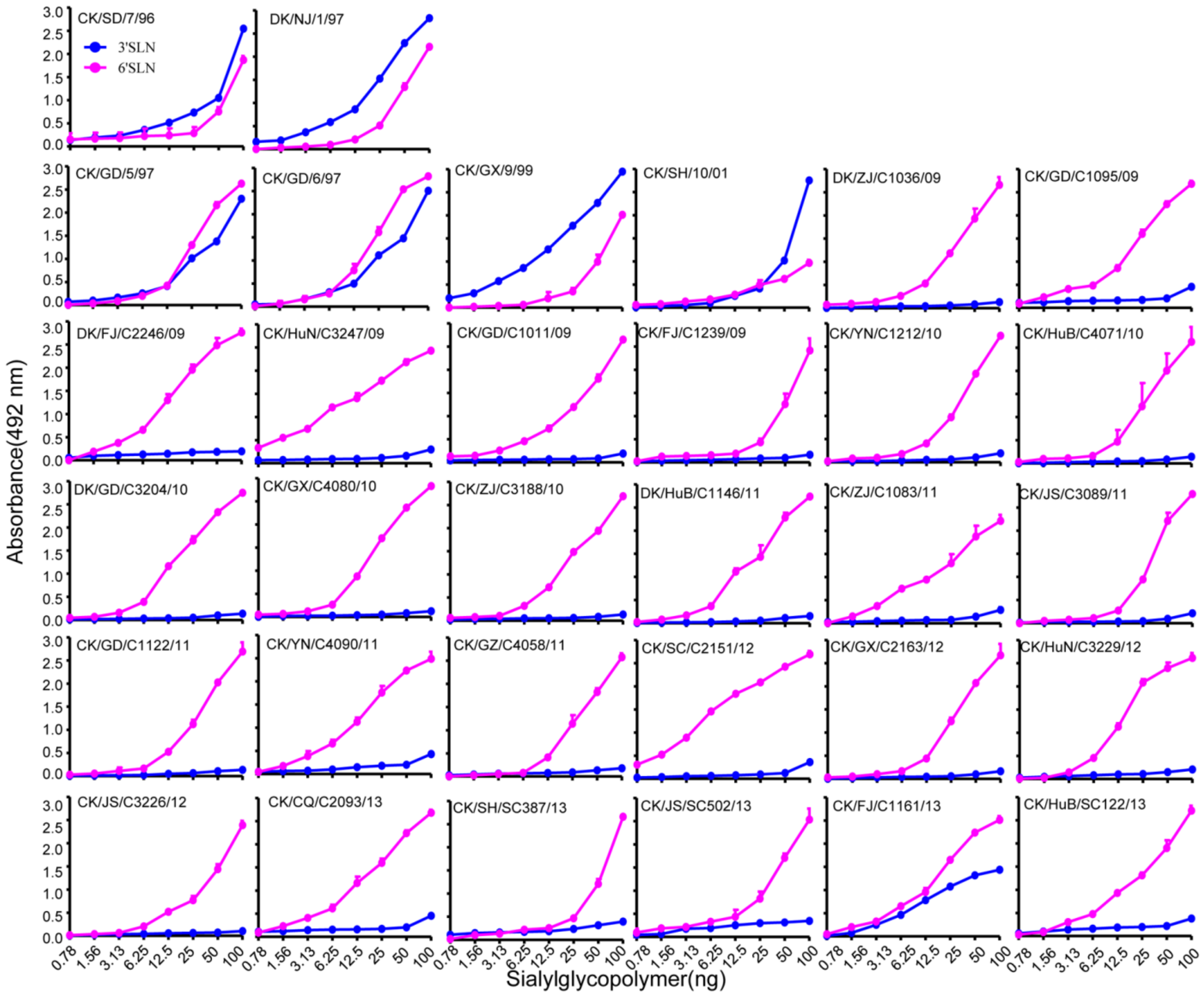

Figure S3. Characterization of the receptor-binding properties of H9N2 viruses. The binding of the viruses to two different biotinylated glycans (α-2, 3 glycan, blue; α-2, 6 glycan, pink) was tested. The data shown are the means of three repeats; the error bars indicate the standard deviations.
