# Supplementary material for: Genetics, Receptor Binding Property, and Transmissibility in Mammals of Naturally Isolated H9N2 Avian Influenza Viruses
Source: PLoS Pathog. 2014 Nov 20;10(11):e1004508. doi: 10.1371/journal.ppat.1004508 (PMC4239090; doi:10.1371/journal.ppat.1004508)
Supplement: Figure S4 — Histological lesions caused by H9N2 viruses in the lungs of ferrets. Ferrets were euthanized on day 4 p.i. with 106EID50 of test virus, and the lungs were collected for pathological study. The lungs of DK/ZJ/C2046/12 virus-inoculated animal showed only mild histopathological changes (H&E staining,) (A), whereas the lungs of CK/HuN/C4136/10 (B), CK/CQ/C1258/11 (C), CK/HuB/C4196/09 (D), CK/ZJ/SC324/13 (E), and CK/SH/SC197/13 (F) virus-inoculated ferrets showed severe pathological lesions (H&E staining). Viral antigen was detected in the epithelial cells of bronchus and alveoli by means of immunohistochemical (IHC) staining (G, from the lung samples of a ferret inoculated with CK/GX/C1435/12 virus; H, from the lung samples of a ferret inoculated with CK/SH/SC197/13 virus). Images A–F were taken at ×100 magnification; images G and H were taken at ×400 and ×200 magnification, respectively. (PDF) [file ppat.1004508.s004.pdf]

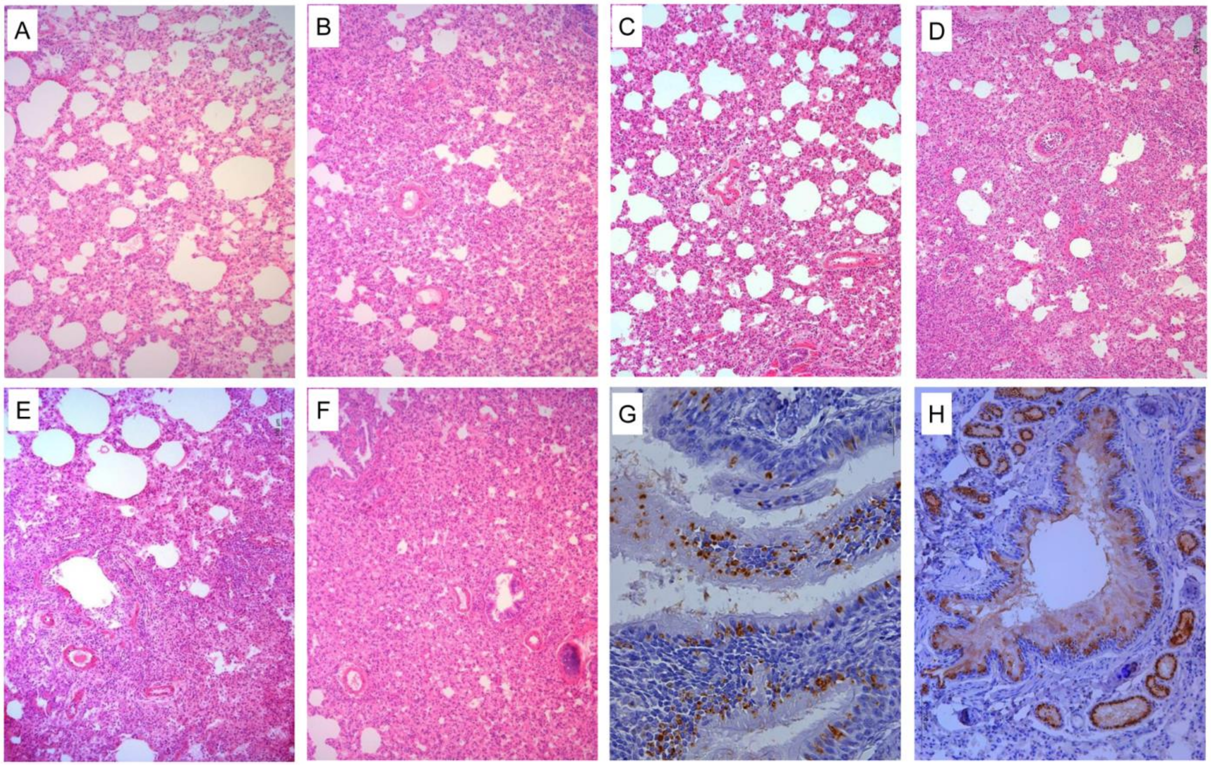

Figure S4. Histological lesions caused by H9N2 viruses in the lungs of ferrets. Ferrets were euthanized on day 4 p.i. with $10^6 EID_{50}$ of test virus, and the lungs were collected for pathological study. The lungs of DK/ZJ/C2046/12 virus-inoculated animal showed only mild histopathological changes (H&E staining,) (**A**), whereas the lungs of CK/HuN/C4136/10 (**B**), CK/CQ/C1258/11 (**C**), CK/HuB/C4196/09 (**D**), CK/ZJ/SC324/13 (**E**), and CK/SH/SC197/13 (**F**) virus-inoculated ferrets showed severe pathological lesions (H&E staining). Viral antigen was detected in the epithelial cells of bronchus and alveoli by means of immunohistochemical (IHC) staining (**G**, from the lung samples of a ferret inoculated with CK/GX/C1435/12 virus; **H**, from the lung samples of a ferret inoculated with CK/SH/SC197/13 virus). Images **A–F** were taken at x100 magnification; images **G** and **H** were taken at x400 and x200 magnification, respectively.
