# Supplementary material for: Genetics, Receptor Binding Property, and Transmissibility in Mammals of Naturally Isolated H9N2 Avian Influenza Viruses
Source: PLoS Pathog. 2014 Nov 20;10(11):e1004508. doi: 10.1371/journal.ppat.1004508 (PMC4239090; doi:10.1371/journal.ppat.1004508)
Supplement: Table S1 — Mutations detected in the H9N2 viruses that contribute to the increased binding to human-type receptors, transmission, replication, and virulence in mammals, as well as to resistance to amantadine and rimantadine. (PDF) [file ppat.1004508.s005.pdf]

Table S1. Mutations detected in the H9N2 viruses that contribute to the increased binding to human-type receptors, transmission, replication, and virulence in mammals, as well as to resistance to amantadine and rimantadine[a].

Virus (Genotype)	HA (H3 Numbering)				PB1				PA	M1			M2	NS1
	I155T	H183N	A190V	Q226L	R207K	I368V	H436Y	M677T	A515T	N30D	T139A	T215A	S31N/G	P42S
DK/ZJ/C1036/09 (1)	T	N	**A**	**L**	K	**V**	Y	T	T	D	**T**	A	**N**	S
CK/GX/C4080/10 (1)	T	N	**A**	**L**	K	**V**	Y	T	T	D	**T**	A	**N**	S
CK/ZJ/C3188/10 (1)	T	N	**V**	**L**	K	**V**	Y	T	T	D	**T**	A	**N**	S
CK/ZJ/C1083/11 (1)	T	N	**V**	**L**	K	**V**	Y	T	T	D	**T**	A	**N**	S
CK/YN/C4090/11 (1)	T	N	**V**	**L**	K	**V**	Y	T	T	D	**T**	A	**N**	S
CK/GX/C1435/12 (1)	T	N	**V**	**L**	K	**V**	Y	T	T	D	**T**	A	**N**	S
CK/JS/C3226/12 (1)	T	N	**A**	**L**	K	**V**	Y	T	T	D	**T**	A	**N**	S
CK/SH/SC197/13 (1)	T	N	**V**	**L**	K	**V**	Y	T	T	D	**T**	A	**N**	S
DK/ZJ/C2046/12 (2)	T	N	**A**	**L**	K	**V**	Y	T	T	D	**T**	A	**N**	S
CK/JS/SC502/13 (2)	T	N	**T**	**L**	K	**V**	Y	T	T	D	**T**	A	**N**	S
CK/HuB/SC122/13 (2)	T	N	**A**	**L**	K	**V**	Y	T	T	D	**T**	A	**N**	S
CK/HuN/C3229/12 (3)	T	N	**V**	**L**	K	**V**	Y	T	T	D	**T**	A	**N**	S
CK/SH/SC387/13 (3)	T	N	**T**	**L**	K	**V**	Y	T	T	D	**T**	A	**N**	S
CK/ZJ/SC324/13 (3)	T	N	**V**	**L**	K	**V**	Y	T	T	D	**T**	A	**N**	S
CK/ZJ/C1219/10 (4)	T	N	**A**	**L**	K	**V**	Y	T	T	D	**T**	A	**N**	S
CK/JS/C4258/12 (4)	T	N	**V**	**L**	K	**V**	Y	T	T	D	**T**	A	**N**	S
CK/JS/C3089/11 (5)	T	N	**A**	**L**	K	**V**	Y	T	T	D	**T**	A	**N**	S
CK/CQ/C1258/11 (6)	T	N	**A**	**L**	K	**I**	Y	T	T	D	**T**	A	**N**	S
CK/CQ/C2093/13 (7)	T	N	**A**	**L**	K	**I**	Y	T	T	D	**A**	A	**N**	S
CK/YN/C1212/10 (8)	T	N	**A**	**L**	K	**V**	Y	T	T	D	**T**	A	**N**	S
CK/GX/C2163/12 (9)	T	N	**A**	**L**	K	**I**	Y	T	T	D	**T**	A	**N**	S
CK/SC/C2151/12 (10)	T	N	**A**	**L**	K	**I**	Y	T	T	D	**T**	A	**N**	S
DK/FJ/C2246/09 (11)	T	N	**A**	**L**	K	**I**	Y	T	T	D	**T**	A	**N**	S
CK/FJ/C1239/09 (11)	T	N	**A**	**L**	K	**I**	Y	T	T	D	**T**	A	**S**	S
DK/HuB/C1146/11 (12)	T	N	**A**	**L**	K	**I**	Y	T	T	D	**T**	A	**S**	S

CK/HuB/C4196/09 (13)	T	N	**V**	**L**	K	**I**	Y	T	T	D	**T**	A	**S**	S
CK/HuN/C3247/09 (13)	T	N	**A**	**L**	K	**I**	Y	T	T	D	**A**	A	**S**	S
CK/HuB/C4071/10 (13)	T	N	**A**	**L**	K	**I**	Y	T	T	D	**T**	A	**S**	S
CK/GZ/C1011/09 (14)	T	N	**A**	**L**	K	**I**	Y	T	T	D	**T**	A	**S**	S
CK/GD/C1095/09 (15)	T	N	**A**	**L**	K	**I**	Y	T	T	D	**T**	A	**S**	S
CK/HuN/C4136/10 (16)	T	N	**A**	**L**	K	**V**	Y	T	T	D	**T**	A	**G**	S
DK/GD/C3204/10 (16)	T	N	**A**	**L**	K	**V**	Y	T	T	D	**T**	A	**N**	S
CK/GD/C1122/11 (16)	T	N	**A**	**L**	K	**V**	Y	T	T	D	**T**	A	**N**	S
CK/GZ/C4058/11 (16)	T	N	**A**	**L**	K	**V**	Y	T	T	D	**T**	A	**N**	S
CK/FJ/S1161/13 (17)	T	N	**A**	**L**	K	**V**	Y	T	T	D	**T**	A	**N**	S
CK/SD/7/96	T	N	**A**	**Q**	K	**V**	Y	T	T	D	**T**	A	**S**	S
DK/NJ/1/97	T	N	**T**	**Q**	K	**V**	Y	T	T	D	**T**	A	**S**	S
CK/GD/5/97	T	N	**V**	**Q**	K	**I**	Y	T	T	D	**T**	A	**S**	S
CK/GD/6/97	T	N	**V**	**Q**	K	**V**	Y	T	T	D	**T**	A	**S**	S
CK/GX/9/99	T	N	**T**	**Q**	K	**V**	Y	T	T	D	**A**	A	**S**	S
CK/SH/10/01	T	N	**A**	**Q**	K	**V**	Y	T	T	D	**T**	A	**S**	S

[a], Mutations that are conserved in the H9N2 viruses are shown in black, mutations that are not conserved in the H9N2 viruses are shown in red.
