# Supplementary material for: Genetics, Receptor Binding Property, and Transmissibility in Mammals of Naturally Isolated H9N2 Avian Influenza Viruses
Source: PLoS Pathog. 2014 Nov 20;10(11):e1004508. doi: 10.1371/journal.ppat.1004508 (PMC4239090; doi:10.1371/journal.ppat.1004508)
Supplement: Table S2 — Virus binding affinity to sialylglycopolymers. (PDF) [file ppat.1004508.s006.pdf]

Table S2. Virus binding affinity to sialylglycopolymers.

Virus	Association constant (K_a, mg^{-1} sialic acid)[a,b]		Relative value[c]
	α 2,6 SA	α 2,3 SA	
CK/SD/7/96	0.73±0.32	2.61±2.55	0.28
DK/NJ/1/97	0.31±0.21	3.48±1.33	0.09
CK/GD/5/97	2.13±0.73	1.28±0.67	1.66
GD/6/97	2.48±0.47	1.32±0.58	1.88
CK/GX/9/99	0.37±0.10	4.54±0.21	0.08
CK/SH/10/01	0.40±0.23	2.19±2.11	0.18
DK/ZJ/C1036/09	2.25±0.96	<0.01	225.71
CK/GX/C4080/10	2.92±0.73	<0.01	292.92
CK/ZJ/C3188/10	2.39±0.49	<0.01	239.10
CK/ZJ/C1083/11	6.30±1.23	<0.01	630.65
CK/YN/C4090/11	6.03±0.56	<0.01	603.56
CK/GX/C1435/12	11.22±6.49	0.10±0.05	112.17
CK/JS/C3226/12	1.43±0.53	<0.01	143.55
CK/SH/SC197/13	3.69±0.00	<0.01	369.47
DK/ZJ/C2046/12	3.93±1.05	<0.01	393.22
CK/JS/SC502/13	1.37±0.32	<0.01	137.53
CK/HuB/SC122/13	5.66±2.46	<0.01	566.76
CK/HuN/C3229/12	6.72±3.32	<0.01	672.81
CK/SH/SC387/13	1.03±0.90	<0.01	103.89
CK/ZJ/SC324/13	2.29±0.58	<0.01	229.38
CK/ZJ/C1219/10	3.75±0.18	<0.01	375.14
CK/JS/C4258/12	5.85±0.61	<0.01	585.47
CK/JS/C3089/11	2.27±1.56	<0.01	227.67
CK/CQ/C1258/11	2.17±0.92	<0.01	217.77
CK/CQ/C2093/13	3.74±1.08	<0.01	374.07
CK/YN/C1212/10	1.81±0.87	<0.01	181.80
CK/GX/C2163/12	1.60±0.20	<0.01	160.21
CK/SC/C2151/12	11.15±2.14	<0.01	1114.94
DK/FJ/C2246/09	7.44±2.46	<0.01	744.99
CK/FJ/C1239/09	1.35±0.91	<0.01	135.95
DK/HuB/C1146/11	3.03±0.52	<0.01	303.93
CK/HuB/C4196/09	2.88±0.86	0.06±0.04	48.02
CK/HuN/C3247/09	10.80±1.69	<0.01	1079.92
CK/HuB/C4071/10	2.06±0.50	<0.01	206.34
CK/GD/C1011/09	2.29±0.67	<0.01	229.11
CK/GD/C1095/09	11.11±5.27	<0.01	1111.47
CK/HuN/C4136/10	2.07±0.55	<0.01	207.18
DK/GD/C3204/10	3.79±0.63	<0.01	379.13
CK/GD/C1122/11	1.36±0.68	<0.01	136.91
CK/GZ/C4058/11	2.56±0.26	<0.01	256.84
CK/FJ/C1161/13	2.38±0.99	1.70±0.63	1.40
rCK/GX/9/99	0.27±0.16	7.54±0.57	0.04
rCK/GX/9/99-HAT155I	<0.01	12.33±1.02	<0.01
rCK/GX/9/99-HAN183H	1.32±0.26	11.88±2.07	0.11

[a] Dose-response curves of virus binding to sialic acid (Sia) were analyzed by using a single site binding algorithm and curve fitting by GraphPad Prism to determine the association constant values (K_a).
[b] Each value is the mean ± SD of three experiments, which were each performed in triplicate.
[c] K_a (α2, 6 Sia)/K_a (α2, 3 Sia), higher relative values reflect higher binding specificity to α2,6 SA.
