# Supplementary material for: Genetics, Receptor Binding Property, and Transmissibility in Mammals of Naturally Isolated H9N2 Avian Influenza Viruses
Source: PLoS Pathog. 2014 Nov 20;10(11):e1004508. doi: 10.1371/journal.ppat.1004508 (PMC4239090; doi:10.1371/journal.ppat.1004508)
Supplement: Table S3 — Virus selection scenarios for the ferret study. (PDF) [file ppat.1004508.s007.pdf]

Table S3. Virus selection scenarios for the ferret study.

Genotype	1	2	3	4	5	6	7	8	9	10	11	12	13	14	15	16	17
Number of viruses	8	3	3	2	1	1	1	1	1	1	2	1	3	1	1	4	1
Number of Provinces in which the virus was detected	6	3	3	2	1	1	1	1	1	1	1	1	2	1	1	3	1
Number of strains selected for the ferret study	2	1	1	2	0	1	0	0	0	0	0	0	1	0	0	1	0
