# Supplementary material for: Genetics, Receptor Binding Property, and Transmissibility in Mammals of Naturally Isolated H9N2 Avian Influenza Viruses
Source: PLoS Pathog. 2014 Nov 20;10(11):e1004508. doi: 10.1371/journal.ppat.1004508 (PMC4239090; doi:10.1371/journal.ppat.1004508)
Supplement: Table S4 — The body temperature increase and body weight loss of each of the ferrets inoculated with or exposed to the different H9N2 influenza viruses. (PDF) [file ppat.1004508.s008.pdf]

Table S4. The body temperature increase and body weight loss of each of the ferrets inoculated with or exposed to the different H9N2 influenza viruses.

Virus<br>(Genotype)	Body temperature increase (℃)		Body weight loss (%)	
	Inoculated	Exposed	Inoculated	Exposed
CK/GX/C1435/12 (1)	1.6, 2, 1.2	3.4, 1.6, 1.6	1.8, 7.8, 7.9	3.2, 0, 5.2
CK/SH/SC197/13 (1)	0.6, 0.8, 1.5	0.3, 1.3, 0.1	0.6, 3.1, 0.4	0, 3.3, 0
DK/ZJ/C2046/12 (2)	2.4, 1.9, 1.8	0, 0, 0	5.2, 3.3, 3.6	0, 2.9, 0
CK/ZJ/SC324/13 (3)	0.5, 1.1, 1.7	0.5, 0.1, 1.3	0.2, 2.6, 2.6	0, 0, 0.9
CK/ZJ/C1219/10 (4)	0.9, 0.7, 0.5	0.2, 1.5, 0	1.2, 1.4, 0	0, 1.8, 0
CK/JS/C4258/12 (4)	1.9, 1.2, 1.5, 0.7, 0.7, 0.9	0.7, 1.5, 0.5, 1.3, 2.4, 0.5	0.8, 2.4, 3.3, 2.5, 1.5, 3.3	6.3, 8.8, 3, 0, 1.2, 0
CK/CQ/C1258/11 (6)	1.2, 2.5, 2.2	0, 0.5, 0	1.3, 1.8, 2.7	0, 0.3, 0
CK/HuB/C4196/09 (13)	1.2, 0.8, 0.7	0, 0, 0	0.5, 1.6, 3.9	0, 0, 0
CK/HuN/C4136/10 (16)	0.8, 1.1, 0.6	0, 0, 0	1.7, 4.2, 3.1	0, 0, 0.5
CK/ZJ/C1219/10-PB2/627K (4)	0.8, 1.4, 1.1	0.1, 1.3, 0.9	1.9, 3.9, 6.4	0, 4.5, 1.1
CK/ZJ/C1219/10-PB2/701N (4)	1.2, 0.7, 1	1.3, 1.1, 0	0, 1.9, 7.2	2.3, 4.4, 0
