# Supplementary material for: Genetics, Receptor Binding Property, and Transmissibility in Mammals of Naturally Isolated H9N2 Avian Influenza Viruses
Source: PLoS Pathog. 2014 Nov 20;10(11):e1004508. doi: 10.1371/journal.ppat.1004508 (PMC4239090; doi:10.1371/journal.ppat.1004508)
Supplement: Table S5 — Assessment of the presence of the 627K and 701N mutations in the PB2 segment of stock viruses and viruses recovered from the nasal washes of H9N2 influenza virus-infected or -exposed ferrets by use of deep sequencing. (PDF) [file ppat.1004508.s009.pdf]

Table S5. Assessment of the presence of the 627K and 701N mutations in the PB2 segment of stock viruses and viruses recovered from the nasal washes of H9N2 influenza virus-infected or -exposed ferrets by use of deep sequencing.

Virus	Stock virus		Virus recovered from animal on different days post-inoculation (p.i.)						
			Animal pair	Day 2 p.i		Day 4 p.i		Day 6 p.i	
	627K	701N		627K	701N	627K	701N	627K	701N
CK/GX/C1435/12	0%	0%	3	13.1%	25.9%	ND	ND	ND	ND
CK/SH/SC197/13	0%	0%	2	ND	ND	ND	ND	0%	0%
DK/ZJ/C2046/12	0%	0%	2	ND	ND	ND	ND	0%	0%
CK/ZJ/SC324/13	0%	0%	3	ND	ND	ND	ND	0%	0%
CK/ZJ/C1219/10	0%	0%	1	ND	ND	ND	ND	5.1%	23.5%
CK/JS/C4258/12	0%	0%	3	0%	7.5%	ND	ND	ND	ND
CK/CQ/C1258/11	0%	0%	1	ND	ND	ND	ND	0%	0%
CK/HuB/C4196/09	0%	0%	3	ND	ND	0%	28.6%	ND	ND
CK/HuN/C4136/10	0%	0%	1	ND	ND	ND	ND	0%	41.3%
